# Gradient in grammatical structure of indigenous languages reflects pathway of human expansion in the Americas

**DOI:** 10.1038/s41598-025-86265-8

**Published:** 2025-04-24

**Authors:** Matthias Urban, Matías Guzmán Naranjo

**Affiliations:** 1 Centre National de la Recherche Scientifique, Laboratoire “Dynamique du Langage” (UMR 5596) & Université Lumière Lyon 2, Lyon, France; 2 University of Freiburg, Freiburg, Germany

**Keywords:** Evolution of language, Social evolution, Social anthropology

## Abstract

The settlement of the Americas is one of the major episodes of prehistoric human dispersal, and involved multiple temporally and geographically uneven demographic events that continued into the Holocene. Here we suggest the possibility that these complex dynamics are reflected in the spatial structure of Indigenous linguistic diversity. On the basis of newly collated data, we find more pronounced spatial structure in linguistic diversity in North America than in South America after known genealogy and language contact are accounted for. Furthermore, we report a continent-wide gradient in aspects of sound systems and grammatical structure that mirrors early north–south dispersal paths, and that is not explained by local language contact and known phylogenetic relationships.

## Introduction

The prehistoric settlement of the Americas is one of the major episodes of human dispersal into previously uninhabited territories. Human population history in the Americas has turned out to be much more complex than previously thought, however. Uncontroversial paleogenomic evidence shows that humans were present south of the Last Glacial Maximum (LGM) continental ice sheets of North America at around 15,000 years BP^1^ (archeological data suggest that humans may have been present further south already much earlier, before and/or during the LGM^2,3^). Population splits leading to the emergence of population structure already occurred among early humans in North America. In the late Pleistocene, a single source population, partly associated with the Clovis archaeological culture, rapidly moved south into South America, possibly in two pulses^1^. Extant demographic, cultural, and linguistic diversity in South America, however, does not only reflect in-situ development of this ancestral population; paleogenomic evidence again shows that there were “multiple independent, geographically uneven migrations into South America”^4^. Such a secondary in-migration event led to a complete population turnover only few millennia after initial colonization. Furthermore, a demographic event impacted the Central Andes after 4200 years BP and involved people with affinities to ancient inhabitants of the California Channel Islands^4,5^; this signal might reflect north-to-south immigration from North America into the Andes; south-to-north migration with an origin point in the Central Andes; or migrations out of Mesoamerica both in a north- and southward direction^6^.

Relatively recent demographic events are in the purview of historical linguistics. The comparative method, the backbone of the field, can establish relationships of common descent that arose several thousands of years ago. In one remarkable application that is relevant in the present context, the Na-Dené languages of northwestern North America have been argued to share a common ancestor with the small Yeniseian language family of Central Siberia^7,8^. This is the first potential demonstration of trans-continental Holocene language relationships. The deeper phylogenetic structure of the remainder of the Indigenous languages of the Americas, however, which belong(ed) to more than 150 different lineages^9^, remain in the dark, and the genesis of the exuberant linguistic diversity of South America is one of the major unresolved questions in historical linguistics.

At the same time, Indigenous languages of the Americas show the linguistic analogue of population structure in how they organize sounds into systems and in the design principles of their grammars. This type of evidence is immaterial to the comparative method (which relies on patterned correspondences between sounds in words and grammatical paradigms that can most economically be explained by common descent). Such population structure can be due to several causes which typically do not occur in mutually exclusive contexts. Possible factors include phylogenetic structure (inheritance within known language families and lineage-specific trends^10^); language contact (the effects of bi- and multilingualism, pervasive in many parts of the Americas); and universal cognitive preferences. Understanding comparative linguistics as a population science^11^, such structured similarities have been argued to make accessible linguistic relationships at a time depth comparable to or exceeding that of the comparative method. This would make them relevant as proxies for late Pleistocene and Holocene dispersal events such as those that took place in the Americas^11–14^.

The case of Na-Dené and Yeniseian is illustrative. The structure of languages of both families is intricately complex; grammatical information is encoded by prefixes to a verb root; grammatical relations such as subject and object are indicated on the verb rather than the noun; the shape of grammatical markers varies in intricate and sometimes unpredictable ways; and some Athabaskan and Yeniseian languages have tonal contrasts. With this profile, Yeniseian languages are unusual in the Eurasian context, where, in more recent history, languages of a type that is radically opposed (belonging to the Uralic, Turkic, Mongolic, and other families) have spread widely. In other words, linguistically, Yeniseian, consistent with its probable phylogenetic affiliation with the New World, can be interpreted as a remnant population that reflects older layers of Siberian linguistic diversity that were for the most part ousted by later language spreads.

Given the multi-factor dynamics of language change and evolution, disentangling the contribution of more recent and deeper local language contact; known phylogenetic structure; universal cognitive preferences; and possibly deep historical affinities –the kind of signal we are interested in here– is an ongoing challenge^15,16^.

Here, we present a model that is designed to identify the contributions of language contact and known phylogeny to observed structural diversity and to isolate signals reflecting historical processes that they do not account for. In addition, our model incorporates information on American population prehistory from paleogenomics, in particular north-to-south dispersals, to model observable linguistic distributions, adding a layer of complexity and realism that to our knowledge so far has not been achieved (this is methodologically and empirically different from an earlier study that claimed “founder effects” in phonological structure that reflect very ancient dispersals Out of Africa^17^).

Rather, our study articulates with and improves on two previous observations on structural variation in the Americas. A direct predecessor study revealed gradient structure in typological variation in western (in particular Andean) South America that extends from Colombia down to Tierra del Fuego and that had not been previously observed in this form^18^. However, this study did not extend to North America, and had no principled way to model regional contact events and phylogenetic structure. Independently, the Americas, in particular North America, have been argued to be divided into two chronologically interpretable linguistic populations^19,20^. One of these, the “Pacific Rim” population, is systematically opposed to a different, pan-American profile. In the most recent interpretation, the Pacific Rim population is interpreted as chronologically older and reflecting the earliest dispersal events down the Pacific coast^21^.

## Results

We surveyed a total of 77 linguistic features that are known to be variable across the Americas in a sample of 102 languages distributed across the continent. Our survey covers basics of phonology (sound structure), morphology (word structure), syntax (sentence structure), and lexicon (structure of the vocabulary); see “Methods” section for more details.

To analyze this dataset, we used the MultivAreate framework^22^. MultivAreate’s approach is based on a multivariate probit structure that allows us to model multiple correlated binary dependent variables with potentially missing observations. One key property of this type of model is that it captures and accounts for correlations between multiple features. Additionally, MultivAreate allows us to assign independent predictors to each individual outcome, or enforce shared parameters across multiple outcomes (see “Methods” for more details). Our MultivAreate model includes two components that account for known phylogenetic and areal structure as well as a component that estimates linguistic distances as a function of known dispersal trajectories in American prehistory. First, we included the late Pleistocene entry of humans into Arctic North America and the subsequent coast-oriented southward dispersal through North America and further into South America. We also included the possible dispersal of people with an ancestry related to ancient People of the Californian Channel Islands to the Central Andes in the mid-Holocene, thus approximating the complexity of demographic prehistory of the Americas as currently understood by paleogenomic studies. This is a significant improvement vis-a-vis previous studies of linguistic distributions and their historical interpretation.

We compared three model specifications. In all three models we include a phylogenetic term and a contact component to account for phylogenetic and contact relationships between the languages. The language classification we relied on for this is that of Glottolog 4.8^23^, which is considered conservative and only accepts phylogenetic structure if well-supported empirically and commonly accepted by specialists.

Model 1 contains a contact component and a phylogenetic term. This is our baseline model, against which we can evaluate whether a further predictor that models prehistoric dispersal vectors has any additional effect once areal preferences and phylogenetic biases are accounted for.

Model 2 contains an additional expansion component based on the distance of each language from Bering Strait along known prehistoric dispersal paths as described above; it is implemented as a linear predictor on the calculated distance.

Finally, Model 3 consists of a phylogenetic term, a contact component, an expansion component, and an additional error term on the distances. Our rationale for designing such a model is that we know that current language locations are unlikely to reflect prehistoric dispersal events directly and exclusively. In a number of cases that is likely significant, languages have reached their present locations through cultural processes of language shift or later language expansions that may be related to demographic processes, but not necessarily those of interest here.

Here, we report results of the most complex model 3 unless specifically noted.

### Overall spatial structure and contributions of each component

To obtain a first general view of the model’s output, we computed the marginal effects for all features based on the spatial structure of the model. To do this we projected a grid of points over the Americas and then predicted the expected value for each point in the grid, based on the model parameters. We carried out this procedure for 500 samples of the model and then extracted the average predictions. Since we have marginal effects for 77 features, we needed some way of compressing them into a single plot which we can interpret. We used the RGB representation technique from spatial dialectometry^24^. We performed Principal Component Analysis on the 77 marginal effects, and mapped the first three components to red, green and blue values of RGB color space. Absolute colors do not have any inherent meaning, but two locations with similar colors are linguistically similar, according to the model.

**Fig. 1:**
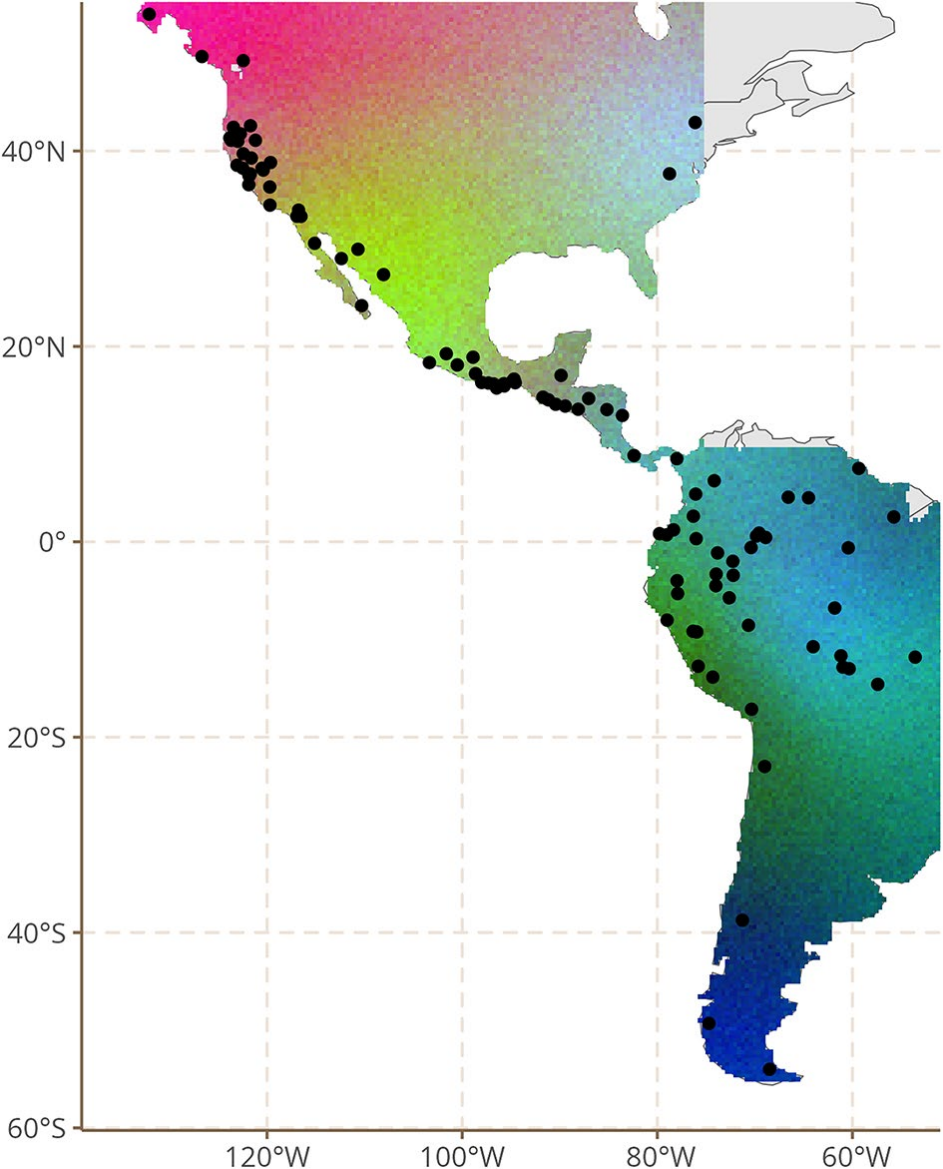
Spatial structure of typological variation in the Americas, based on PCA of marginal effects of linguistic features mapped onto RGB color space. This map was made using ggplot2^25^, and sf^26,27^.

Figure 1 shows the spatial structure of typological variation in the Americas in this way (Supplementary Material 1 shows maps for all models). In general, the visual impression is one of gradual rather than abrupt changes in structural profiles of Indigenous languages, in particular in the western part of the continent (we discuss this phenomenon at length further on). In some cases, transitions seem to be more abrupt, e.g. in Mesoamerica. At the same time, however, we also observe structural affinities between discontinuous parts of the Americas. This is salient for Eastern North America (though interpretation must be cautious given the low sample density), Amazonia, Patagonia and Tierra del Fuego.

Comparing these results with the qualitative literature we observe that the contact component of our model picks up the signals of known linguistic areas identified in the literature. We discuss six of these—the Pacific Northwest Coast, Northern California, Mesoamerica, the Andes, Amazonia, and Patagonia– in Supplementary Material 2.

We also explored how our model picks up phylogenetic signals in the data. These were clearest for recent language families and became gradually weaker the older the language families are judged to be in the literature (impressionistically or on the basis of quantitative analysis). For the oldest language families that may have begun to develop up to 5000 BP, intra-family diversity was not significantly different from diversity in the sample as a whole. We discuss the results relating to known phylogenetic structure in more detail in Supplementary Material 3.

Finally, we explored the estimated error term concerning present-day language locations (Supplementary Material 4). Overall, the model does not find very large likely discrepancies between the calculated distances based on historically attested or present-day locations and what it estimates to be more accurate distances. We caution against too strong inferences from this observation; however, one possible interpretation is that, when investigated across diverse language families, language locations in the Americas in many cases have not been affected significantly by post-dispersal demographic events related to the expansion of known language families.

### High structural diversity in North America

**Fig. 2:**
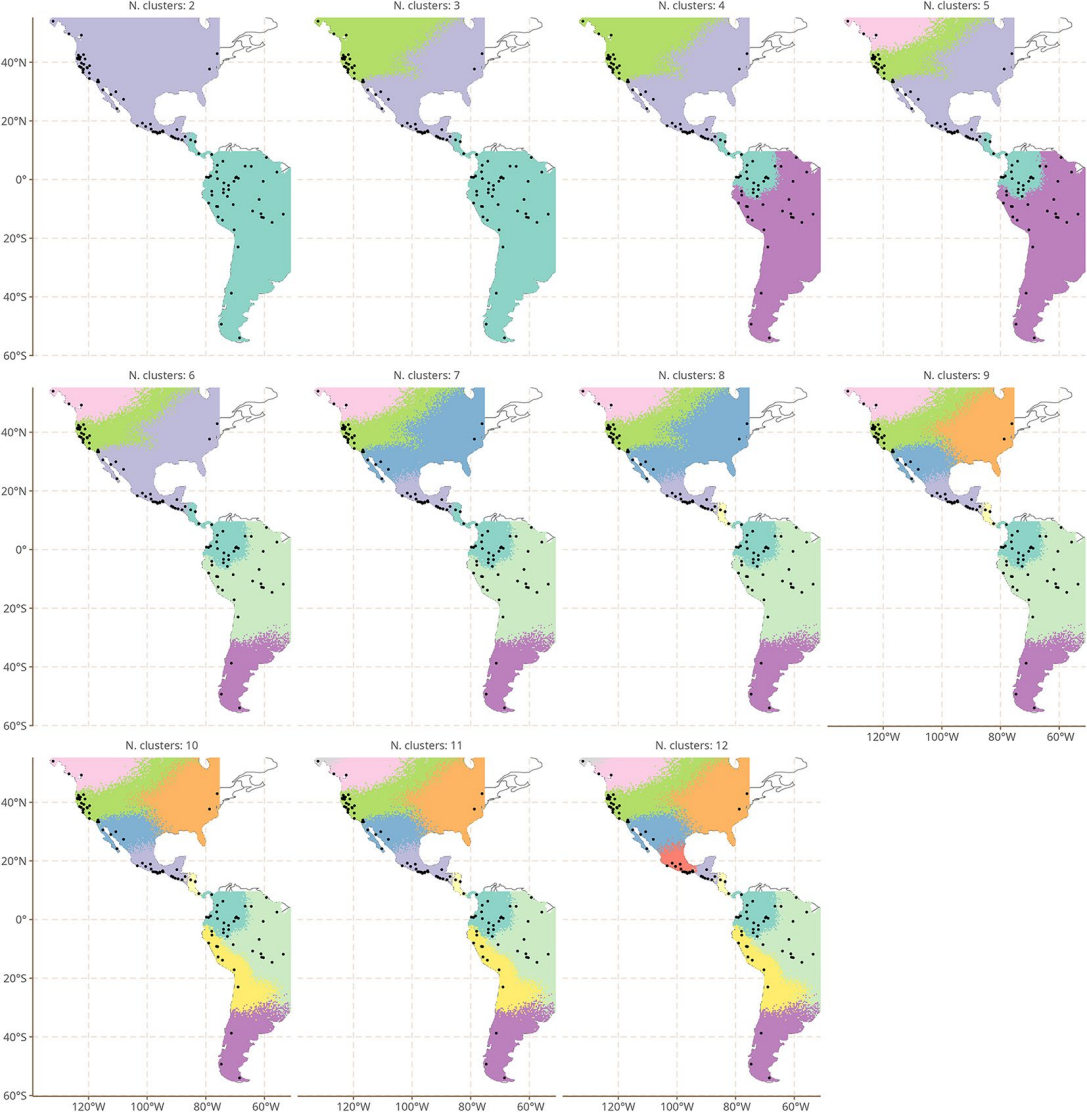
Hierarchical clustering on the spatial marginal effects. This map was made using ggplot2^25^, and sf^26,27^.

Figure 2 shows a hierarchical clustering algorithm applied to the spatial marginal effects of Model 3. We began by first enforcing a minimum number of two clusters and then stepwise increased the number of clusters to 11.

The basic split at n = 2 is between North and South America, but already at n = 3 clusters, we see the Pacific Northwest Coast languages distinguished from the rest of North America. We discuss the dynamics of the Northwest Coast linguistic area, and the deep origins of the linguistic similarities between families found there, in Supplementary Material 2.

More generally, we note that up to n = 5, clusters are repeatedly distinguished only within North America, pointing to overall greater diversity in North America than in South America after phylogeny and contact are taken into account. This result is at first glance surprising, as South America is viewed as highly diverse typologically; our results suggest that this impression partially reflects the impact of known phylogenetic structure and areal convergence on observed diversity.

## Latitudinal gradients in feature distributions

### Coefficients and marginal effects of the linear component

**Fig. 3:**
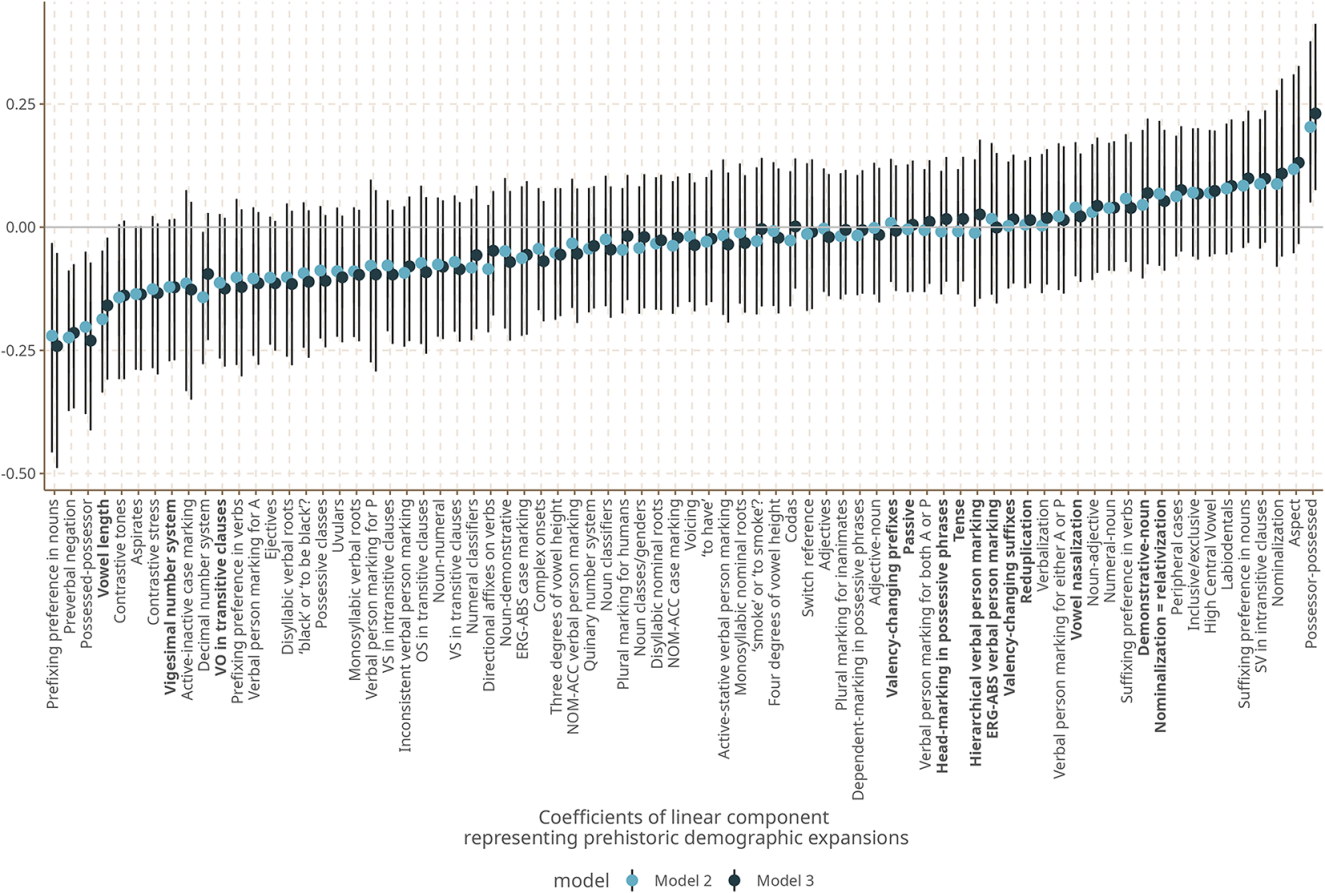
Estimates for the linear component of Model 2 and Model 3. Features in bold are more stable^28^.

For each of the features we survey, Fig. 3 shows the coefficients of the linear component representing the north–south direction of prehistoric demographic expansion.

For many features, the effect of the position of language communities along the southward dispersal paths (plus, where appropriate, the closest distance to that path) are either close to zero or completely overlapping zero. Overall, the estimates obtained from Model 2 and Model 3 are very close to Model 1, showing that the expansion component only has a small impact on feature estimation. However, for several features the posterior does *not* significantly overlap with zero, indicating that for a subset of the features in our dataset, the model finds a clear linear effect of prehistoric dispersal pathways on the probability of observing certain values.

Furthermore, the marginal effect of the expansion component is relatively large for several of the features in question. While most features have relatively high uncertainty, the component’s impact on the estimated probability to observe a certain feature state is notable for several features, with a change of up to 0.5 probability between the northernmost point of known expansion paths (i.e. close to the inferred entry point of humans near Bering Strait) and the southernmost point in Tierra del Fuego.

The strongest effects are observed for a number of heterogeneous features of sound patterns and grammatical structure. This set includes the position of the word or affix that negates a clause before or after the verb; the relative ordering of possessor and possessed as exemplified e.g. by English *John’s house* vs. Spanish *la casa de Juan*; the indexation of grammatical information on nouns by prefixes as opposed to suffixes; and the existence of long vs. short vowels.

**Fig. 4:**
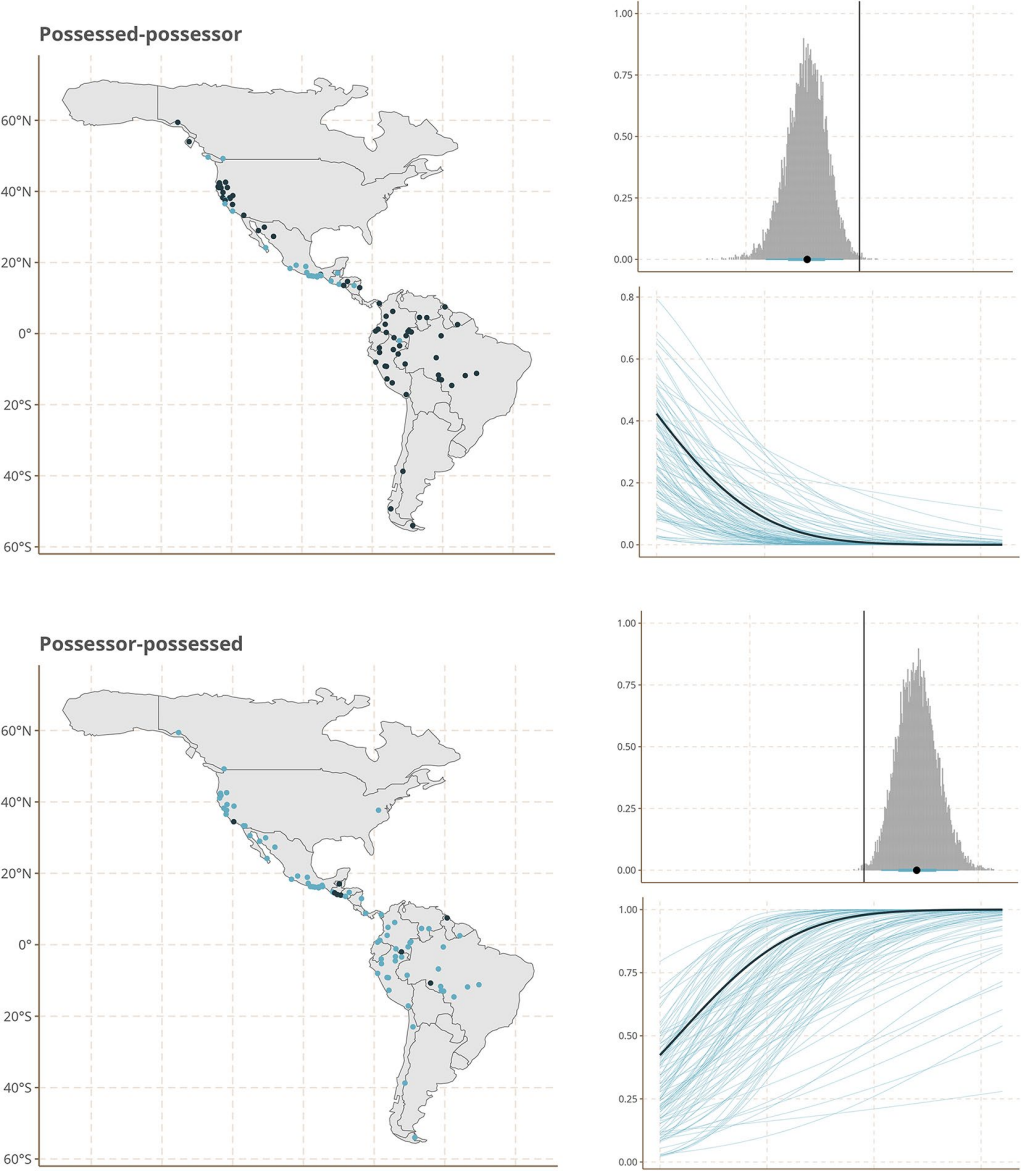

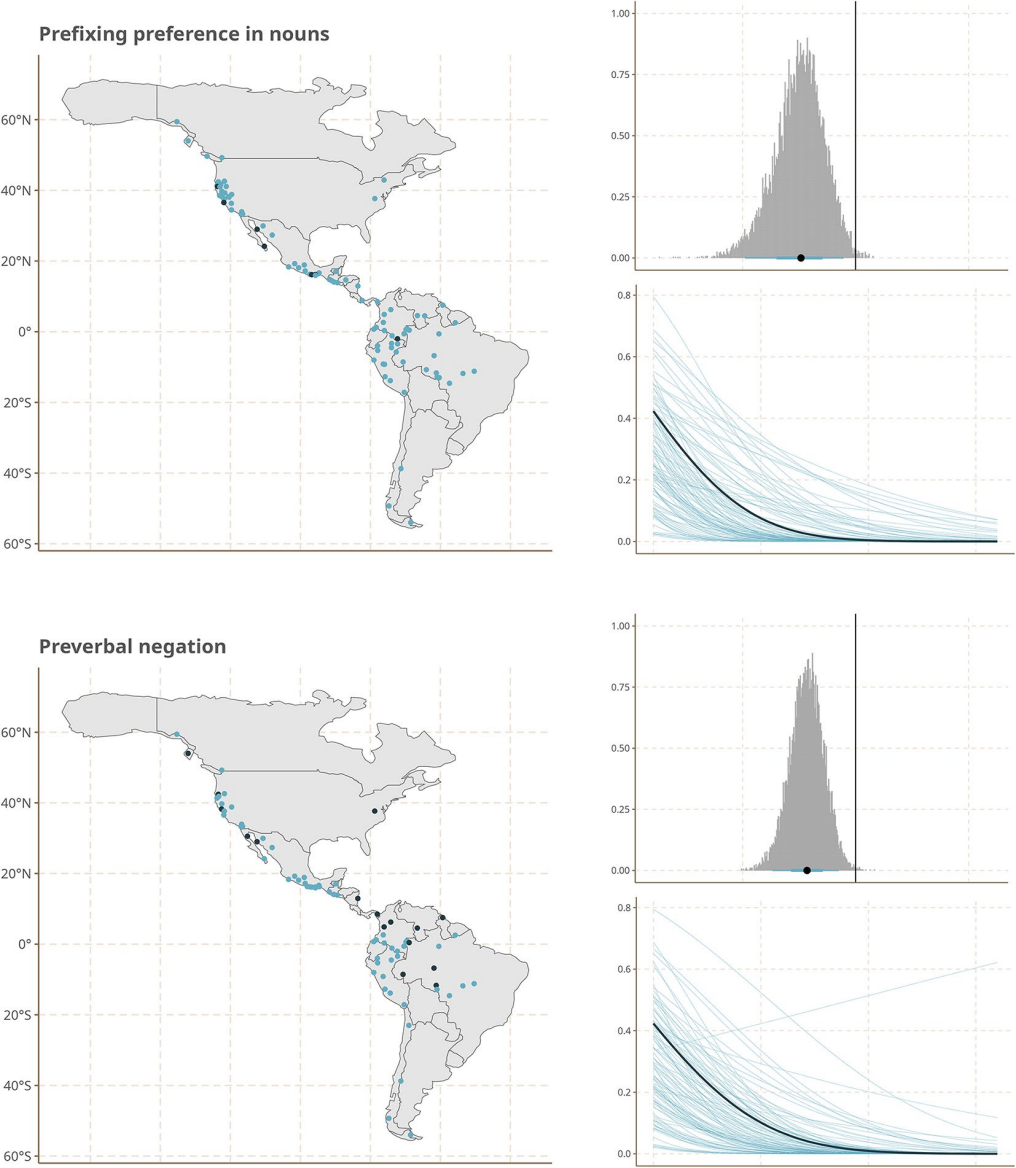

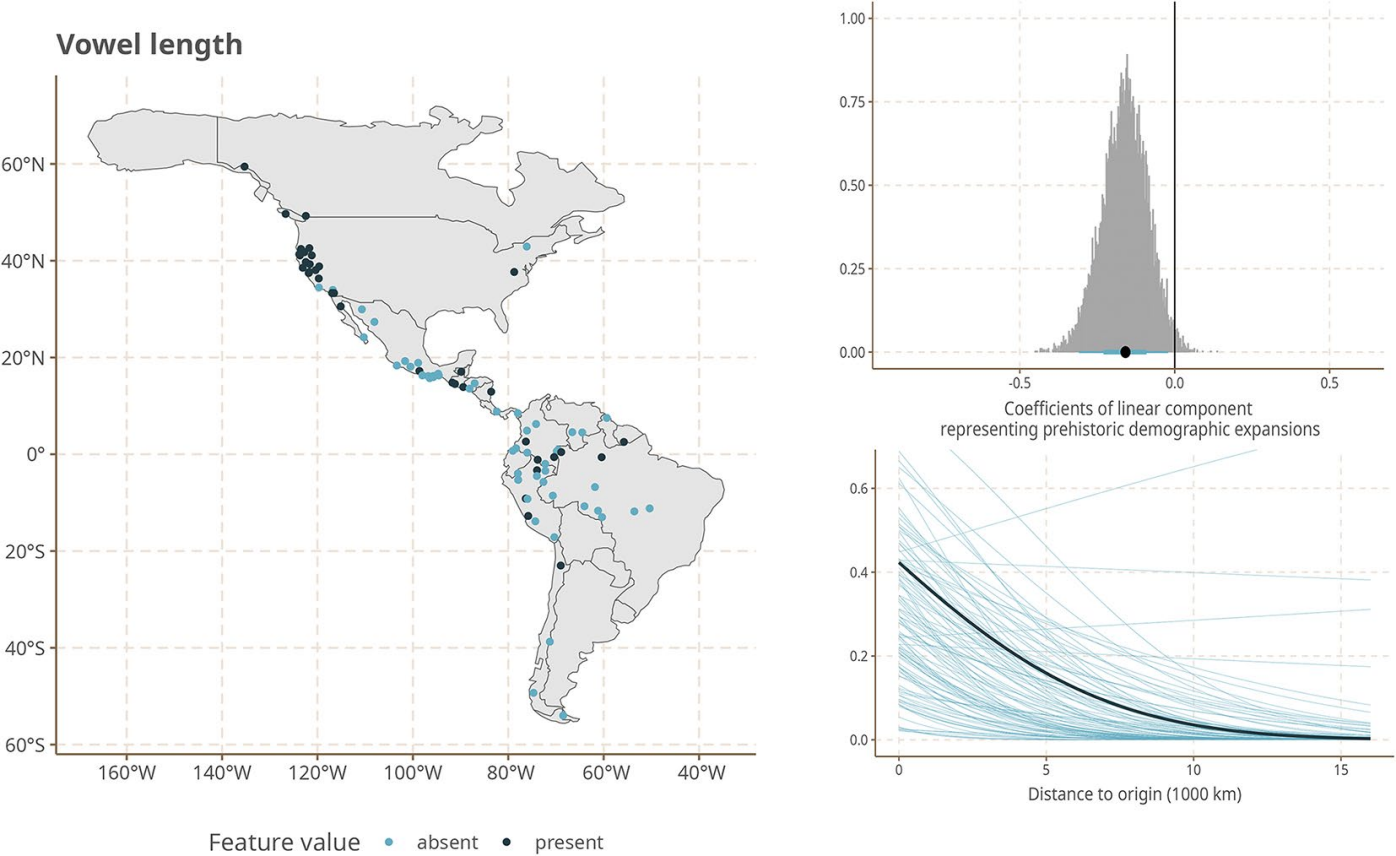
Geographical distribution of Features states (left), marginal effects (top right) and coefficients (bottom right) of the linear component representing prehistoric demographic expansions for five features with the largest coefficients. Figures created using ggplot2^25^, and sf^26,27^.

To make this more palpable, we show the geographic distribution of the states of these features in our sample together with effect size from our Model 3 in Fig. 4.

While the visual impression of the distribution of the feature states on the maps is indeed one of gradual change of frequencies from north to south, we emphasize that the gradient structure that we infer cannot simply be read of the maps. Instead, it is estimated by our model while taking into account linguistic areality due to cultural contacts and known phylogenetic structure of the languages in our sample.

Furthermore, while gradient structure is particularly pronounced for the five features shown in Fig. 4, it is not the case that they are the only ones that contribute to our model results. In fact, while these features taken by themselves do not point to a single module of phonology or grammar that might track language history, once we look at the full set of features and their effect sizes, we see that they represent three broader areas of phonological and grammatical organization: thus, the strong marginal effect observed for a prefixing preference in nouns is mirrored by a smaller one for the prefixing of verbs in the same direction, and the effect for the order of possessed and possessor is mirrored by other features with relatively strong marginal effects that also relate to the order of syntactic constituents. In addition, features describing consonant systems, in particular the presence of certain types of marked (i.e. cross-linguistically rare) consonants (aspirates, ejectives, uvulars) have relatively high effect sizes. Supplementary Material 5 contains a figure that shows the effect sizes for all features we survey.

**Fig. 5:**
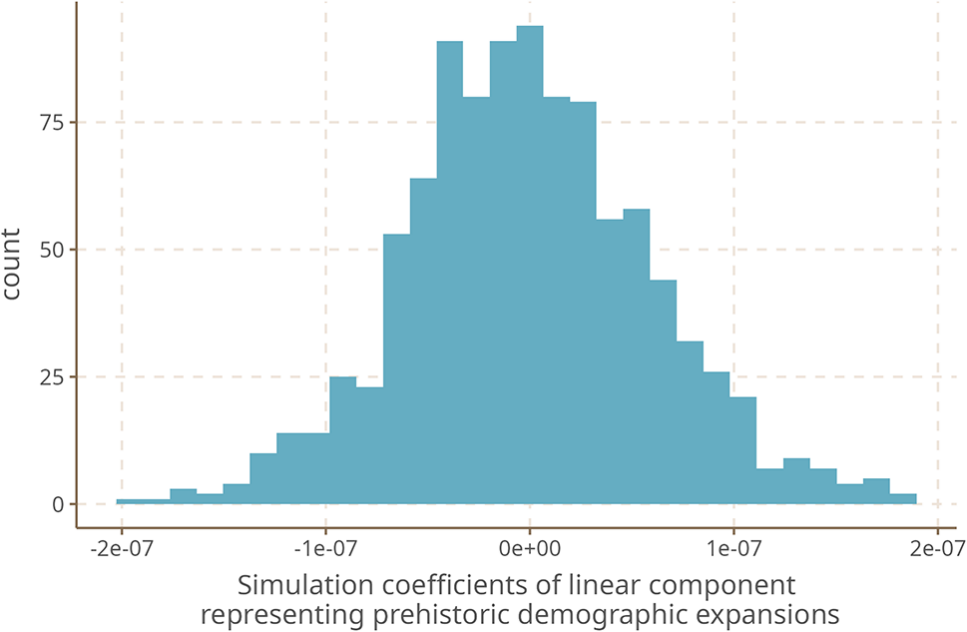
Distribution of the coefficients of 10,000 logistic models on randomly generated data.

Our dataset includes a large number of features, each of which we assessed separately. While known dependencies between features in our case do not significantly reduce the informativeness of each feature (Supplementary Material 6), there still is a very limited design space, and many features are highly plastic^29,30^. For both reasons, we might expect a spatial correlation of some feature values with trajectories of demographic expansion just by chance, similarly to a false positive in frequentist statistics; the absence of a single coherent area of phonology and grammatical structure that the most relevant features would point to might likewise suggest that some of the results are spurious. While it is difficult to definitely show that this is not the case, we performed a simulation to assess this under some specific assumptions. For the purpose of the simulation, we assumed that the contact and phylogenetic components of our model are working perfectly as intended, and that they account for all similarities due to common descent and contact. Under this assumption, we tested whether randomly generated binary variables over 102 observations correlate with distance to the entry point of humans into the Americas along expansion trajectories by fitting a logistic model predicting the observations from that distance. We repeated this process 10,000 times. The distribution of the coefficients is shown in Fig. 5. We observe that the largest effects have sizes of about 2e-7, which is about 10,000 times weaker than most of the effects we found in the actually observed data.

To further assess the robustness of this result, we performed a second analysis using a more traditional approach involving regression modeling with group-level effects for language area and phylogeny. We applied this to both our dataset and another large-scale typological database that has not been designed to capture variation in languages of the Americas, and observed a weak trend for longitudinally structured typological variation (see Supplementary Material 7 for details).

We also analyzed typological variation in Eurasia and attempted to model it as distance from a fictive entry point in the west, again using standard typological databases. We obtained miniscule effect sizes (Supplementary Material 7); this is consistent with the simulation study for the Americas that suggests that we should not observe even the small number of features that we identify as carrying a longitudinally structured gradient signal.

### Model evaluation and model comparison

We then turned to an evaluation of the performance of the three successively more complex models as a whole. To do this, we performed tenfold cross-validation (CV). Cross-validation consists of splitting the data into 10 groups of observations. We trained the model leaving one of the groups out, and then used the trained model to predict the left-out group of observations. We did this in two ways. In the first cross-validation approach (CV-1), we split the data into 10 groups by language, i.e. we excluded all observations for each of the left-out languages. In the second approach (CV-2), we left 10% of all observations out, but ensured that for all languages at least some observations are included. We implemented this by re-coding the left-out observations as missing data. The advantage of this approach is that the model can use the correlation structure across features to infer the missing data points. This allows the spatial component and the expansion component to access information about all languages because we at no point leave out a whole language. In the CV-1 approach, we leave whole languages out, which means that the model is missing information for the spatial and expansion components. We therefore expected that CV-2 should produce better results than CV-1. In particular, we expected that this approach would allow the model to produce better estimates for the error term in Model 3, since all languages have at least some observations. Both approaches to model comparison and model performance are not impacted by issues of model complexity. When predicting unseen data, more complex models are only better than simpler models if the added complexity captures some real pattern in the data. This means that we do not expect the more complex model to perform better unless the added complexity is justified by the data.

The mean balanced accuracy (i.e. the accuracy taking into account class imbalances) of CV-1 and CV-2 for each model is shown in Table 1. While all three models achieve higher than random predictive power, the models which include a dispersal effect performed slightly better overall than the model which does not, and the model which estimates a potential error rate on the distance of language locations to dispersal trajectories had higher accuracy than the model which uses the distances based directly on present locations of the languages. Even under cross-validation, estimating an error on the distances thus helps the model perform somewhat better.

**Table 1:** Mean balanced accuracy for the three models.

Model	CV-1 Balanced accuracy	CV-2 Balanced accuracy
Model 1	0.541	0.543
Model 2	0.549	0.547
Model 3	0.55	0.554

**Fig. 6:**
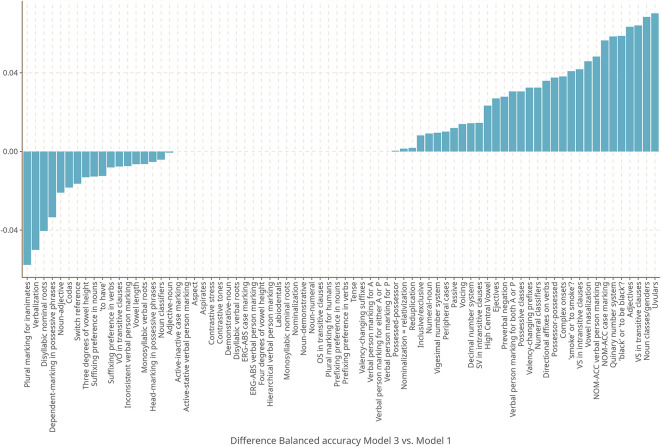
Difference in balanced accuracy by feature between Model 3 and 1.

Figure 6 provides a visual impression of the difference in balanced accuracy by feature between the most complex Model 3 and the baseline Model 1. It is apparent that Model 3 not only outperforms Model 1 in many more features (32) than Model 1 outperforms Model 3 (18), but also that it does so by a larger margin. This shows that the expansion component and the uncertainty parameter on the expansion component help the model predict new data. A more thorough feature by feature comparison including Model 2 can be found in the supplementary materials.

### Secondary expansions

We also tried to model the recently detected Holocene spread of Channel Island-related ancestry in California, Mesoamerica, and the Central Andes^4–6^. To implement this, we adopted a method to asymmetric linguistic contact effects (described in more detail in the “Methods” section). However, assuming a secondary north-to-south dispersal from California to the Central Andes (which, as reviewed in the introduction, is not the only scenario consistent with the paleogenomic data at this point), we could not observe a linguistic correlate in the model’s output, and when included in a larger model the performance degraded considerably in terms of effective sample size and divergences.

## Discussion

Incorporating information on pathways of prehistoric north–south dispersal in the human colonization of the Americas leads to a better model of structural feature distributions in currently or recently spoken Indigenous languages of the Americas when compared with a model that only accounts for known phylogenies and regional language contact effects.

This observation can be interpreted in different, not necessarily mutually exclusive ways, and there is no default interpretation of such signals that can be derived from qualitative knowledge on language history and language change. We therefore provide extended discussion of our results and their possible interpretation in the following.

As we have noted, a general challenge in modeling feature distributions in linguistics is to disentangle the signals of inheritance within known language phylogenies; the influences stemming from the interaction of speakers of neighboring languages; and universal preferences. When contact effects are not explicitly modeled, the cumulative effect of local convergence can produce continent-wide typological structure^15,31^, including large-scale gradients across geographical axes^32^. Localized contact events are captured by the Gaussian Process of our model [Supplementary Materials 2], but we cannot assume that this works perfectly. However, even if such a residue contributed to our results, we would know of no identifiable mechanism related to language contact and areality that could plausibly account for spatial structure on the pan-continental scale on which we observe it: there is no archaeologically visible agent or other social, political, or economic structure that could have brought people into contact over such long distances (the most relevant candidate are supposed maritime trade networks linking Ecuador with other parts of the Pacific Coast and Western Mexico in late prehistory^33,34^; however, most of the languages spoken in the relevant coastal regions have ceased to be spoken, and analysis of data from those languages that survived long enough to be documented at least to some extent are inconclusive^18^). Similarly, we cannot link the observed gradient pattern to large-scale diffusion of crops or other socioeconomic innovations etc. Also signals of known genealogies are explicitly modeled in our approach. As we show in the Supplementary Materials 3, families do not always maintain a consistent profile and intra-family variation increases with the age of the family. The oldest families, such as Arawakan, which may have begun to diversify around 5,000 years ago, show a heterogeneous profile and have levels of internal variation that are not appreciably different from the sample as a whole.

Large-scale longitudinal gradients in language structure are against general expectations derived from studies on the large-scale geography of linguistic feature distributions. One such study shows that linguistic features spread more easily on latitudinal axes as a result of demographic processes related to the expansion of known language families or cultural contact^32^. Longitude, on the other hand, tends to inhibit the spread of features as a concomitant of such processes. However, in our case, we still observe a clinal distribution of features precisely along the unexpected longitudinal axis, and that even when taking into account spatial proximity (as a proxy for contact between speakers of neighboring languages) and phylogeny as part of our model structure.

As our ability to control for known phylogenies and cultural contact in linguistic data has increased dramatically in recent years, a suite of recent studies suggest deep-time correspondence scenarios between phonological and grammatical structure and population history^12,13,16^. Remarkably, such historical signals would have been preserved even in the presence of significant noise added by confounding factors that arose as known linguistic areas and lineages formed and evolved.

To explore whether such an interpretation is consistent with the particulars of our specific case, we return to the results of our cluster analysis as a starting point. Here, we have observed that structural diversity in North America that cannot be accounted for by known phylogenies and contact exceeds that of South America. To the best of our knowledge, this has not been observed in this form so far; however it is generally consistent with the longer presence of humans in North America that implies a longer time frame for in situ differentiation of languages entering with early waves of colonization (and dovetails with the historical linguistic principle that, other things being equal, areas of greatest diversity are likely those in which diversification has had the longest time to operate^35^).

Our results are also suggestive of chronological layers in the structural linguistic diversity in Western North America: Eastern North American languages show greater affinities with the languages of western Mexico (from which they are only separated at the 9th iteration of the algorithm) than with those of California and the Northwest Coast. The Pacific Northwest languages are in fact the first to be distinguished from the remainder of North- and South American languages, which is indicative of profound differences. Both observations suggest a dynamic in which additional pools of diversity successively came into being in Northwest North America with weaker or, for some languages, even no structural links to the East of the continent. This in fact is the case: the Northwest Coast cluster includes Tlingit, a basal language of the Na-Dené family which has likely linguistic connections to Eurasia^7^ and is thought to have arrived in America only in the mid-Holocene^36^ (the cluster also includes languages whose lineages have been hypothesized, but not proven, to have a similar background of secondary in-migration^8^). We also consider it possible that the excessively large error estimates our model returns for the geographical location of the Na-Dené languages (Supplementary material 8), which indicates difficulties fitting these languages into the geographical context given their typological profile, may reflect a linguistic history that is different from the remainder of the Indigenous languages of the Americas. This history is consistent with a secondary genomic contribution of Siberian (“Paleo-Eskimo”) ancestry in Arctic and Northwest Coast people, including those of Na-Dené and Eskimo-Aleut linguistic affiliation (the latter of which not included in our studies), in the same time frame^1,37^ (however the exact demographic connection between Yeniseian and Na-Dené is not obvious:^38,39^).

Thus, where demographic and linguistic events are closest to the present and hence most clearly traceable, some including by traditional comparative methods of historical linguistics, we observe that structural and genealogical diversity patterns in ways that are congruent with vectors of specific prehistoric dispersals into North America.

This makes it possible to hypothesize that the latitudinal spatial gradient we observe across the entire Americas is a reflection of the same or qualitatively similar population dynamics at deeper time depths and greater geographical scales. This would be consistent also with the frequently ancient character of features defining linguistic areas in the Americas (Supplementary material 2), which often reconstruct to the ancestral languages that gave rise to known lineages and whose origins therefore transcend the time horizon of traditional methods.

**Fig. 7:**
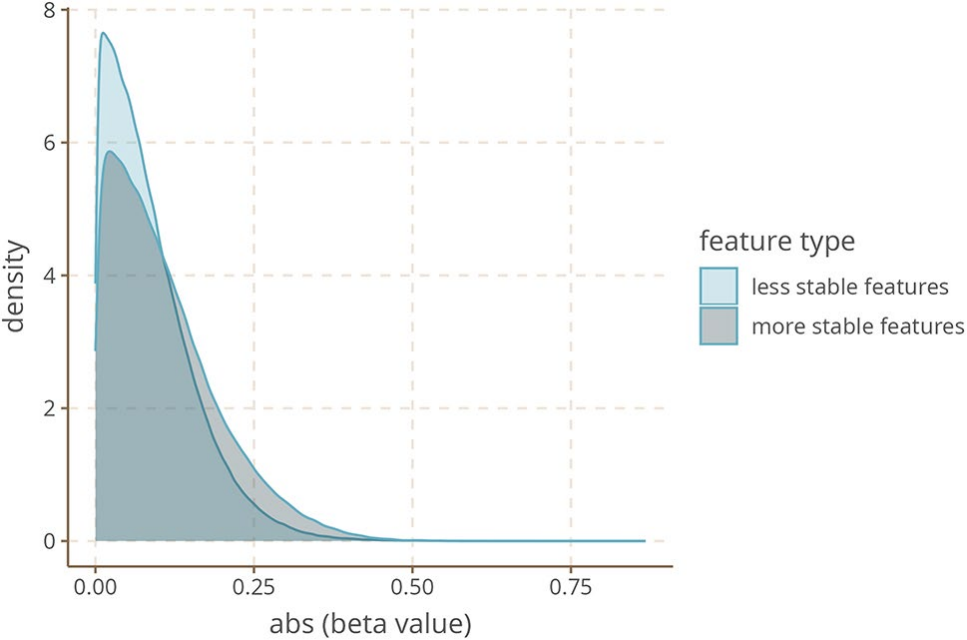
Conditional effects plot of log normal model testing feature stability as a predictor of effect size as DTO coefficients.

To look into this possibility from a further perspective, we decided to investigate the relative stability of individual linguistic features covered in our survey and how their observed feature values relate to geography, in particular north–south dispersal effects. While there is still much uncertainty associated with the topic, a number of design features of languages are thought to be relatively resistant to change as languages develop. A meta-analysis of different methods to identify such stable properties of linguistic structure has found considerable agreement between them^14^. Under an interpretation of the spatial structure across the Americas as reflecting early demographic history, we would expect that more stable features should show gradient spatial variation more strongly than unstable ones. Therefore, we looked at the 20 most stable features identified by meta-analysis^14^ that have analogues in our dataset. We observe a very small difference between stable and non-stable features, to the effect that more stable features have slightly higher mean effect size (0.11) than less stable features (0.09). If we compare these means in a log-normal model, it turns out that stable features have about a 0.25 (95% uncertainty interval: 0.24–0.26) larger mean effect size. Alternatively, we can ask what portion of the posterior of more stable and less stable features overlap with each other. We find that the overlap of more stable and less stable features in Model 3 is 0.79, and in Model 2 0.83 (Fig. 7).

Overall, there is a very small difference between more stable and less stable features; feature stability may contribute to some, though limited, extent to explaining why some features show a more clear expansion effect than others. This does not lend decisive support for a “deep time” interpretation, but is also not inconsistent with it.

Precisely such a clinal distribution of linguistic features in terms of deep human prehistory has been proposed before. Specifically, it was argued that phonological complexity levels have undergone processes analogous to founder effects as humans expanded out of Africa^17^. However, the present case would be significantly different from this. We would not have to assume the logic of founder effects at all. All we observe is that values of features differ in a spatially structured way along a north–south axis reflecting dispersal paths, with no implications as to different complexity levels. The features themselves would be specific as historical markers of linguistic history of the Americas^40^, and reflect selectively neutral linguistic evolution (cf.^41^ on ejectives) that unfolded as present-day observable linguistic diversity was configured; the vicissitudes of language evolution in other areas would most probably lead to other features emerging as diagnostic. Furthermore, the scenario we are considering is different in that we look at variation across entire grammars, not just crude counts of phoneme inventories, and in that we apply a sophisticated model that simultaneously handles contact effects and phylogenetic structure, and identifies the clinal structures when the effects of these are taken into account. Finally, we are concerned with time depths that are much more plausibly reflected in linguistic distributions than the Out-of-Africa dispersal in very early human prehistory.

This study is not ultimately conclusive, but strengthens the general plot that sound patterns and grammatical structure might be cultural indicators of ancient population history in several parts of the world^11,13,14,16,19,24,40^, and have led to longitudinally oriented spatial structure of linguistic diversity in the specific case of the Americas^11,19,21^. Independently of the concrete details, such an interpretation would fit well with an emerging consensus among scholars and native peoples that Indigenous connections to lands and places across the Americas go back far in time and have strong and enduring historical roots.

However, our study has several limitations. Several of these concern the dataset: we would ideally want to include more languages from Eastern North America as well as Eskimo-Aleut languages into our analysis; and we would like to model Paleosiberian languages as an outgroup to more firmly demonstrate, rather than stipulate informally, the historical signal in structural data linking Na-Dené, and, as we suspect, also Eskimo-Aleut languages, with their continent of origin.

We also do not have enough information on the history of the relevant design features of Indigenous languages of the Americas in the time frames accessible to standard methods of historical linguistics and areal typology. As far as we are aware, the only feature with strong effect size in our study that has been investigated in some detail is postverbal negation, a cross-linguistically rare preference for negators to follow rather than precede verbs in South America^42^. In this particular case, an early tilt in frequency distributions towards postverbal position of negators may have created a self-reinforcing mesh of areal pressure that maintains and reinforces the preference through grammaticalization processes that constantly add new conforming structures to the pool of negation strategies^42,43^. This may provide a glimpse at the relevant *longue durée* language dynamics behind gradient feature distributions such as those we observe, and exemplify how the dynamics of the broader linguistic ecology on large scales is linked to internal language change. Richer and more comprehensive qualitative perspectives on this and other relevant features and their evolution, however, might affect and alter the interpretation decisively.

Finally, we have no principled explanation for why we should observe a pan-continental gradient that most plausibly would reflect early pan-continental dispersal processes, but no clear signal for significantly later mid-Holocene demographic events that led to the presence of Channel-Island related ancestry in the Central Andes (however, the points of origin and vector of the underlying demographic processes in this particular case are currently still far from clear, and it may be that the north-to-south dispersal of people from California to the Central Andes that we assumed here is not how this paleogenomic signal actually arose^6^).

## Methods

### Data extraction

**Fig. 8:**
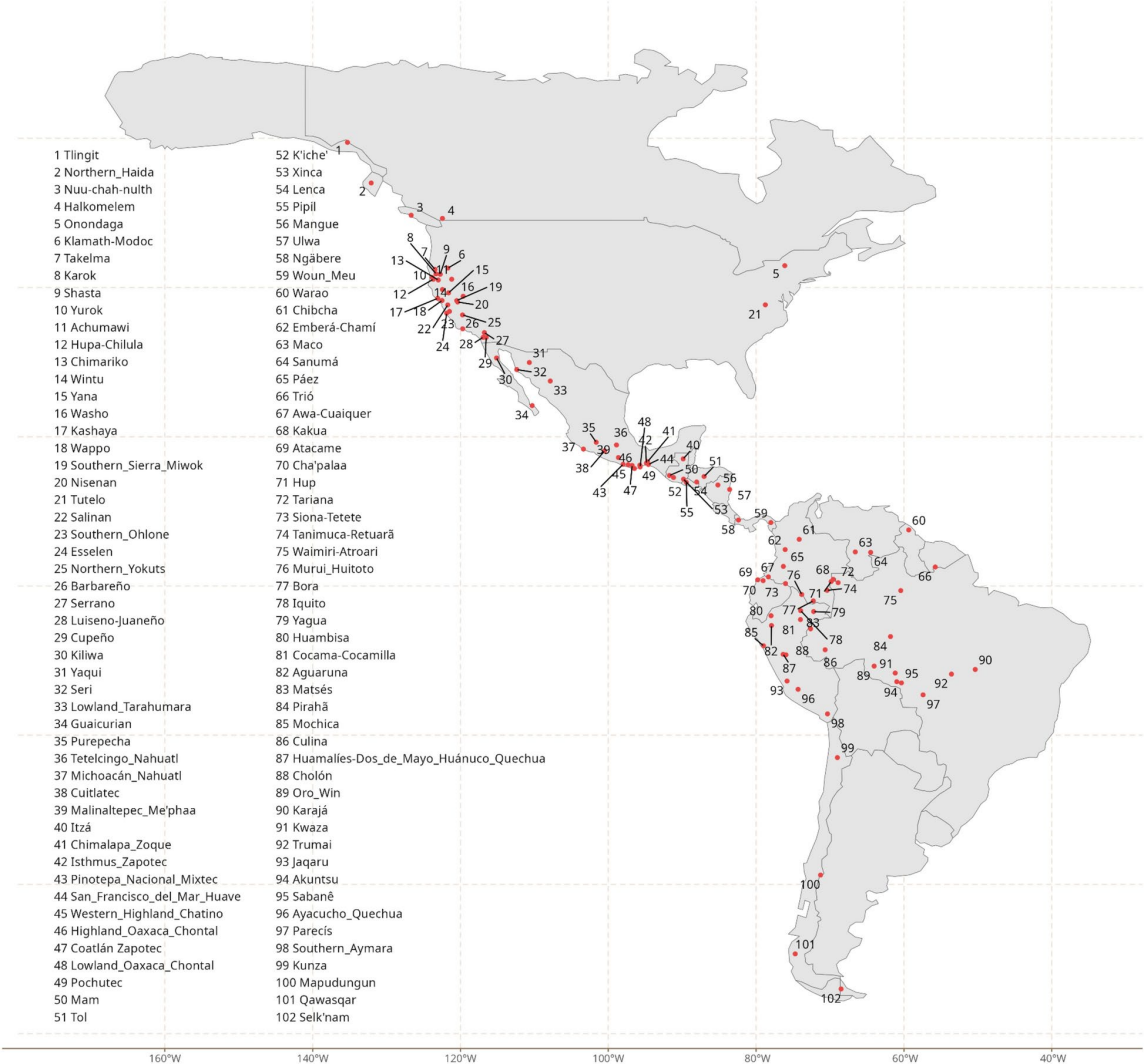
Languages in our sample. This map was made using ggplot2^25^, ggrepel^46^ and sf^26,27^.

We analyzed data on the sound (phonological), grammatical (morphosyntactic), and lexical (vocabulary) structure of 102 Indigenous languages of the Americas. Data come from primary descriptions composed between the 17th to the twenty-first centuries (the oldest colonial sources have been re-interpreted by modern scholars according to current descriptive practices). We sample the Pacific coast, which is of special relevance for prehistoric dispersals in the Americas, particularly densely. However, we also include a number of languages from the western part of North and South America in a way that is commensurate with the genealogical and typological diversity of these regions. Amazonia is hyperdiverse both in terms of the number of linguistic lineages as well as typological structure, whereas North America east of the Rockies hosts a comparably more modest number of languages and lineages with broadly similar structural characteristics. Therefore, we included a significantly larger number of Amazonian languages, whereas eastern North American languages are represented by languages belonging to two of three widespread lineages. Another way to think of the sample design is that it reflects not only our interest in the Pacific, but also the fact that in North America, linguistic diversity is centered in the west, whereas in South America it is centered more in the east, specifically greater Amazonia. Figure 8 plots the location of the sampled languages.

Data partly come from a previous study^18^, though for the majority of languages (n = 58) they have been gathered specifically for the analysis here; a complete list is in Supplementary Material 8. The selection of features, unlike standard worldwide databases^44,45^, has two properties that improve their suitability to explore the questions we wish to address: first, they cover traits that are actually known to be variable in the Americas (though not necessarily in any predefined pattern), limiting the number of features that are likely uninformative. Second, the selection of features privileges ones that can be coded also for languages that are no longer spoken and are poorly and incompletely documented. This is crucial, for many of the languages that were spoken in key regions on the Pacific coast belong to this group, and their testimony would otherwise remain inaccessible for systematic analysis.

Typological traits are frequently interdependent, and such correlations have also been reported for some of the features surveyed in our dataset. For instance, in languages in which the object of a sentence preferably precedes its verb, there tend to be postpositions, whereas prepositions are preferred in languages in which the order of object and verb is the opposite^47^. In addition, there are some logical dependencies that arise when a positive value for one feature excludes the possibility of a positive value for another. For instance, if a language has no plural marking at all, it also does not have plural marking for human nouns. In our model, however, there were no strong residual correlations between features once contact and phylogeny were taken into account. More information can be found in Supplementary Material 6.

### Inter-rater reliability assessment

Since data were extracted by different individuals at different levels of experience, we have conducted an analysis of inter-rater reliability. Two datasets, for a total of 154 datapoints, were coded by the two coders who were responsible for the majority of the data. Both were using the same sources, and were using the instructions for coding specified in the questionnaire. They did not receive any further more specific instructions. Comparisons show that coders most frequently disagree in whether, based on the consulted source(s), a feature can be assigned a specific value with the required level of certainty at all, or if the feature should better be left uncoded (i.e., coded as NA). Coders judged this differently in 19 cases, i.e. 12.3% of datapoints. Where both coders agreed that a feature can be coded based on the source, agreement was notably higher, and they disagreed only in 13 cases, or 8.4% of all datapoints. These percentages are lower than in a recent large-scale database of linguistic structure that is directly comparable in nature and in the way it was assembled^45^; basic disagreement on codability is even more than twice as low.

### Topographic distance calculation

We use topographic distances to build the contact and expansion terms in our model^48^. Topographic distances are the shortest distances between two points, but respecting elevation changes in the terrain. We use the gdistance package^49^ to calculate all topographic paths. For computational reasons, we used a point in the Darien region in northern Colombia as a choke point, meaning we could calculate topographic distances in North America and South America separately, and then combine them. In this paper we measure distances exclusively via terrestrial routes, even though southward expansions, including early ones, may have taken place by watercraft^50^. While Paleoindians possessed seafaring skills that allowed them to colonize islands near the Pacific coastline^51^, direct evidence for Paleoindian seafaring is unavailable, and it is unclear how big the role of maritime routes was in the colonization of the Americas. However, any movement on water would likely have been largely parallel, and close to, the vectors of land-based routes down the Pacific Coast, so that we would not expect significantly different distances when allowing for maritime routes.

### Distance to Bering strait along dispersal trajectories

To implement our knowledge about prehistoric dispersal paths and subsequent demographic events, we first built a set of topographic paths along dispersal trajectories discussed in the most recent review article at the point of writing^1^. Since the locations of most languages do not fall directly onto these dispersal paths, for each language, we calculated the topographic distance to the closest point on the paths. For the analysis that considers the initial dispersal of human populations into the Americas from Beringia, distances are calculated for each language as the sum of the distance from the point of entry along the reconstructed dispersal path plus the distance of the language’s location to the closest point on the dispersal path.

### MultivAreate (Multivariate probit regression with shared spatial component)

The main model we use in this paper, MultivAreate, estimates areal effects in linguistic data^22^. At its core, this model is a multivariate probit regression. In such a model multiple binary dependent variables, which may contain missing data and which may be correlated, can be modeled simultaneously. We do this by assuming that all outcome variables come from a latent multivariate normal distribution. In MultivAreate we can have individual and separate predictors for each dependent variable, or share predictors across some or all dependent variables. Parameter sharing is a way to control for bias in any single predictor, as the parameter estimates will regularize to conform to all predictors.

We present three models. Model 1 has a phylogenetic term and a spatial/contact term. Model 2 has a phylogenetic term, a spatial/contact term, and a linear expansion term based on the distance of the area in which our sampled languages are spoken from the point of entry along dispersal paths as described above. Model 3 has the same terms as Model 2, with an additional (strictly positive) measurement error term on the distance to expansion paths.

We assume a single spatial covariance matrix for all outcome variables, but all other terms are independent for each outcome variable. All models were fit using Stan^52^. We ensured that all Effective Sample Sizes were high enough for inference, and that there were no divergent transitions. Mildly informative priors were chosen based on domain knowledge and computational considerations.

### Phylogenetic regression

To control for phylogenetic relationships between the languages in our sample we include a phylogenetic regression term^53,54^. In a phylogenetic regression we add group effects (also called random effects) for each language in our sample, but force the estimates to be correlated according to a phylogenetic tree^55^. In this work we use the Glottolog tree as a starting point, and calculate branch lengths using lexical data^56,57^

### Gaussian process

To capture contact and non-linear spatial dependencies between our languages we use a Gaussian Process^58–60^. A Gaussian Process takes a distance matrix and transforms it into a covariance matrix using a kernel^59^. The model uses the covariance matrix to sample from a multivariate normal distribution which captures the fact that languages which are spoken in the same region are more similar than languages which are further apart. The key aspect is that the spatial covariance decay is non-linear. While two languages which are spoken in nearby regions can have high spatial covariance, languages that are spoken in distant parts of the Americas can have a covariance of effectively zero.

### Measurement error estimate

This component is an innovation of our approach vis-a-vis other work on the history of structural feature distributions and their interpretation in comparative linguistics. We included this component because we know that the current locations of the languages in our sample do not accurately represent the total distance “traveled” by the people that speak those languages and their ancestors. In other words, there is a high degree of measurement error in our computations when we use current locations for the languages in our sample directly as the basis of the calculations. To account for this we estimated a potential error rate for geographical distance from the dispersal trajectory. We allowed the model to estimate an error term parameter for each language (sampled from a half-cauchy distribution), and calculated the linear expansion effect on a new variable equal to the distance from Bering Strait via the prehistoric dispersal trajectories plus the error term.

### Secondary expansions

To test secondary expansions, we used a method designed to capture asymmetric contact effects in linguistics^60^. For this method, we divided our data into source and target languages. The source languages are those found in regions from which we expect secondary expansions to have started, and target languages are those which could exhibit a reflex from the secondary expansions (see “Results” section for discussion of the problematic nature of this assumption). We started by fitting a model predicting the feature values of the source languages using a Gaussian Process, and then calculate the posterior expected values (Z) of the target languages. Z represents the expected effect of the source languages on the target languages, assuming modern day locations and dispersion based on a simple Gaussian Process model. We then predict the feature values of the target languages from Z. Unlike a simple contact model, this type of model is not limited by the feature values of the target languages when estimating the effects of the source languages on the target languages. Known secondary expansions cover very large distances. To account for this, we used very wide length-scale priors on the Gaussian Process. In a scenario in which languages like those spoken in the regions we modeled as staging areas of secondary expansions had some influence on the structure of languages in the target of these dispersals, or if they replaced them, we would expect that Z should be a good to moderately good predictor of the structure of the target languages. In none of our experiments were we able to find any such effect with this approach.

## Supplementary Information

Supplementary Information.

## Data Availability

All the data and code we used can be accessed at: https://osf.io/2mcy5/?view_only=7331656febd14f45a5666901e5100d6f
