# Supplementary material for: Gradient in grammatical structure of indigenous languages reflects pathway of human expansion in the Americas
Source: Sci Rep. 2025 Apr 24;15:14365. doi: 10.1038/s41598-025-86265-8 (PMC12022089; doi:10.1038/s41598-025-86265-8)
Supplement: Supplementary file 1 — Supplementary Information. [file 41598_2025_86265_MOESM1_ESM.docx]

# Supplementary material 1: Visual representation of marginal effects for additional models


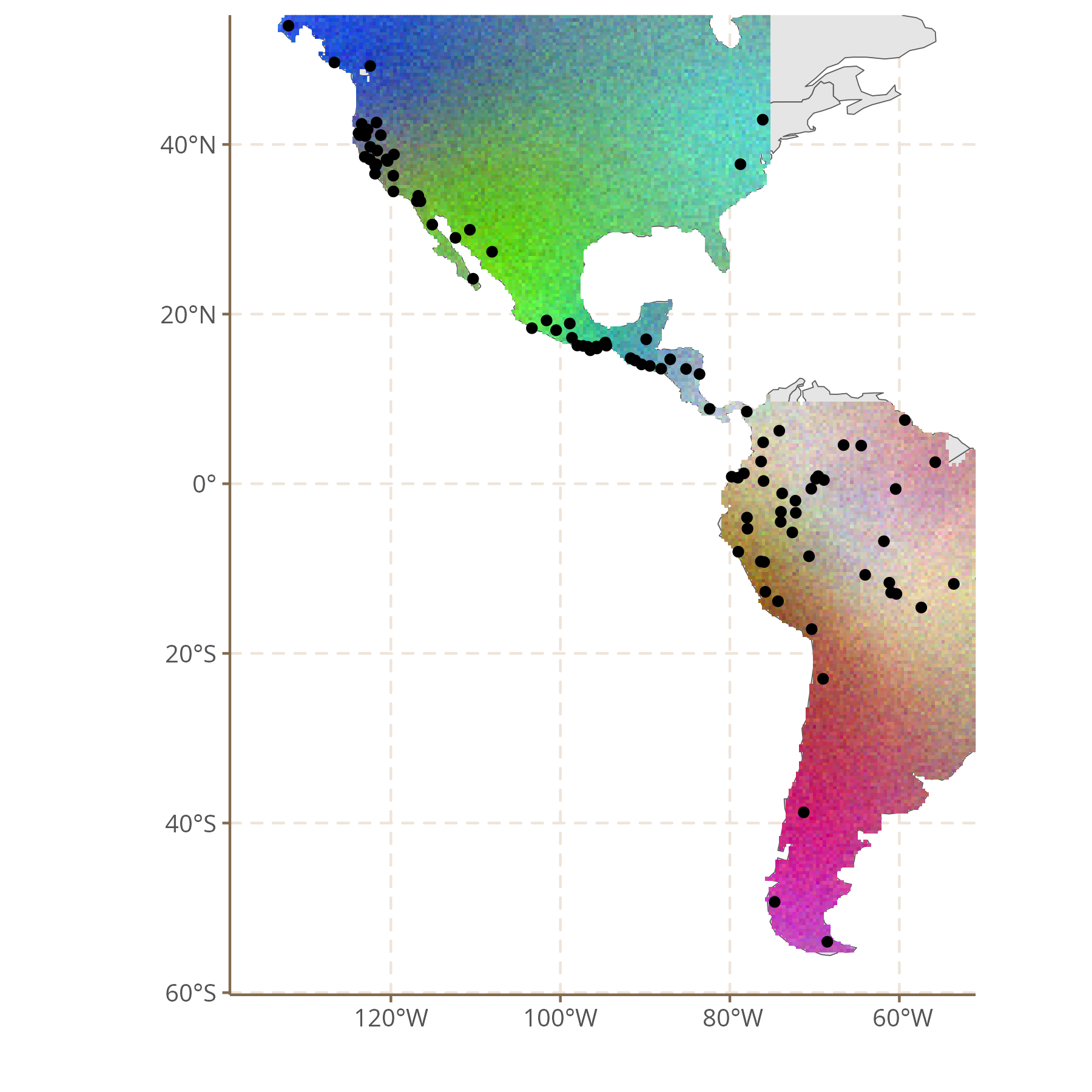


Figure S1.1. Spatial structure of typological variation in the Americas, Model 1, based on PCA of marginal effects on linguistic features mapped onto RGB color space. This map was made using ggplot2 (Wickham 2016), and sf (Pebesma 2018, 2023).


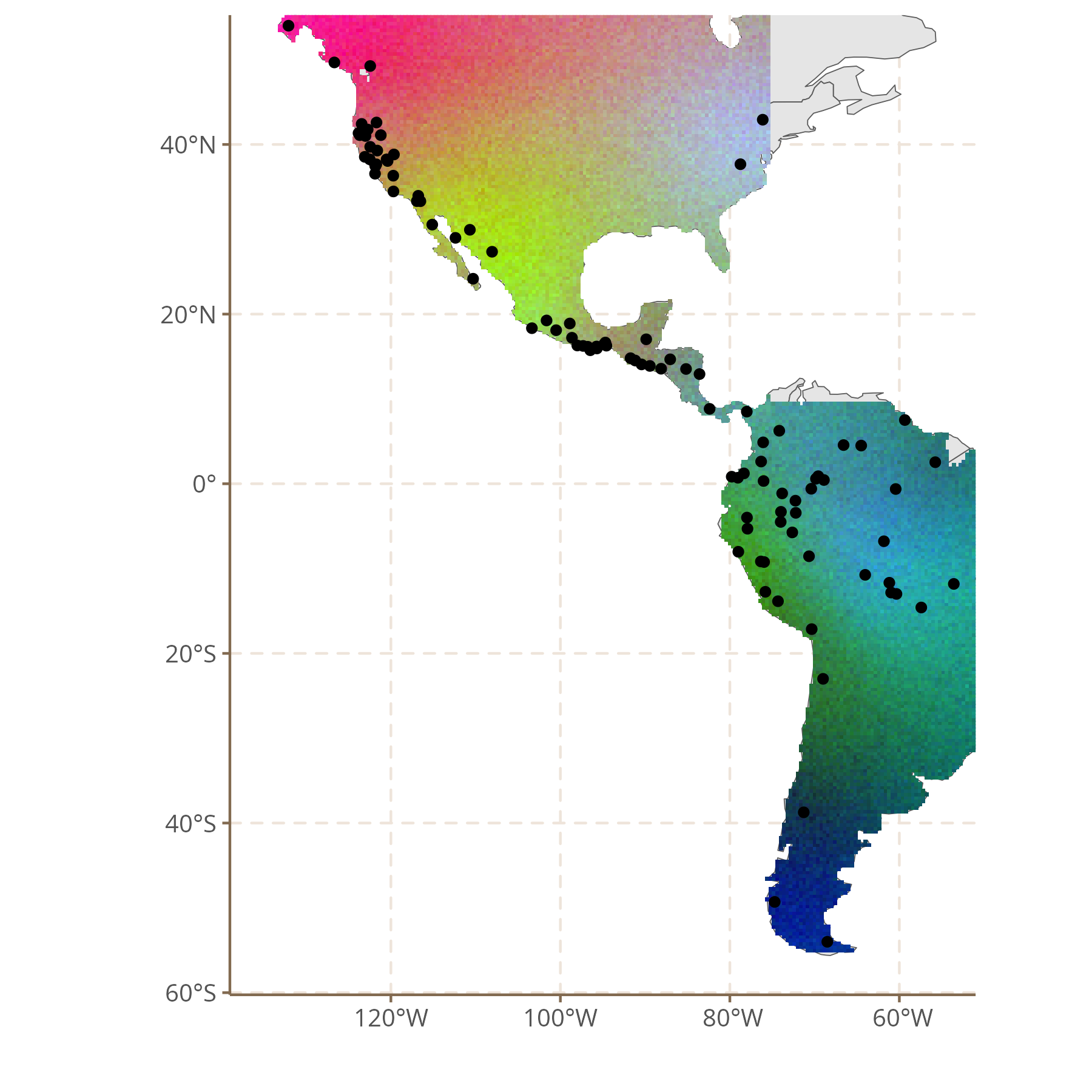


Figure S1.2. Spatial structure of typological variation in the Americas, Model 2, based on PCA of marginal effects on linguistic features mapped onto RGB color space. This map was made using ggplot2 (Wickham 2016), and sf (Pebesma 2018, 2023).


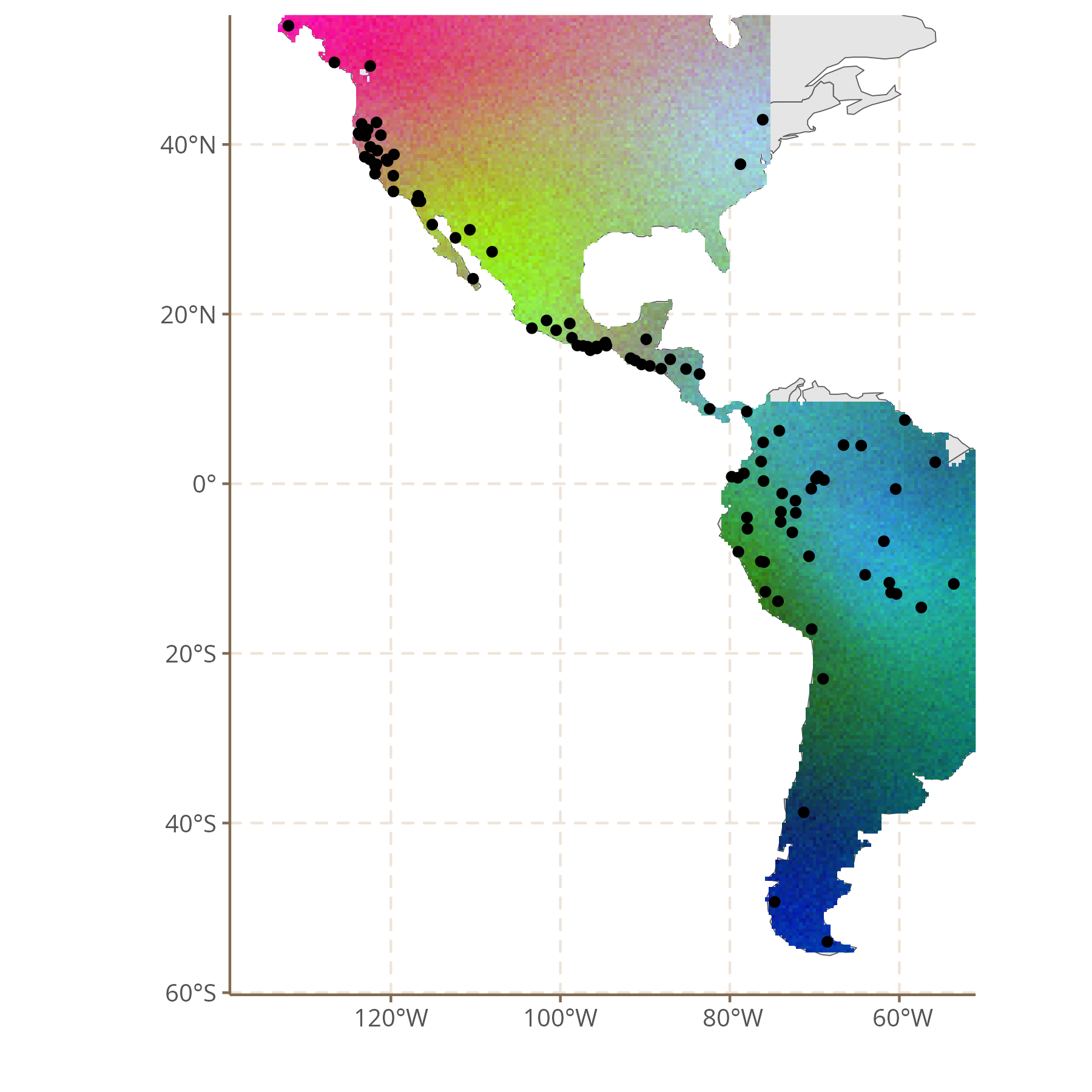


Figure S1.3. Spatial structure of typological variation in the Americas, Model 3, based on PCA of marginal effects on linguistic features mapped onto RGB color space. This map was made using ggplot2 (Wickham 2016), and sf (Pebesma 2018, 2023).

# Supplementary material 2: The contact component and known areality

As part of our exploration of the model results, we looked at the different components separately as a baseline to assess the verisimiltude of the model output. We started with the contact component, and used qualitative research that has sought to establish contact-induced, areal structure in linguistic distributions in the Americas as our standard of comparison.

Traditionally, linguists have posited so-called linguistic areas or “Sprachbünde”. These are regions in which unrelated or not closely related languages have come to share aspects of how their sound inventories are structured and how their grammars function as the result of interaction between their speakers.

Relevant research typically aims to identify language areas on the basis of shared features due to linguistic convergence (very explicitly e.g. Beck 2000 on the Pacific Northwest). However, decades of research have shown that different language contact scenarios can yield very different outcomes in the lexical and grammatical systems of involved languages. In particular, languages may also *diverge* under contact (Evans 2018) as speakers seek to maintain linguistic difference as an index of social difference. We cannot detect such cases by means of our contact component; even though cases of contact-induced divergence may exist in the Americas, we are not aware of evidence for this mentioned in the qualitative literature on the most salient proposed linguistic areas (which we survey below).

On another cautionary note, proposed linguistic areas can only manifest themselves in our result in the values of the features we survey. These pertain to grammatical organization, but also include some aspects of phonological and lexical organization. Many of the features we survey are likely below the awareness threshold of speakers, making it unlikely that they consciously manipulate them. If that assumption is correct, the signals we pick up are likely to have originated in pivot-matching (Matras and Sakel 2007) between structures and constructions on behalf of fully bi- or multlingual speakers to a large extent, and are less likely to result from more ephemeral contact situations with limited bilingualism. Thus, the types of contact situations that would be reflected in our data is likely constrained by the particular features we survey.

Most importantly for our purposes, there are inherent difficulties with the notion of linguistic areas. As reified entities, they have proven extremely difficult to delimit and define consistently and objectively [Campbell 2017]. In fact, while a core area can relatively easily be defined, the literature frequently reflects an uncertainty when it comes to delimiting the geographic boundaries of linguistic areas. As a result, more recently linguistics have moved away from reified, neatly delimited areas to describing the distribution of features in space [Muysken 2008], as we in fact do, too. While we do recognize the footprint of proposed linguistic areas in our dataset, we also see the abovementioned characteristic reflected in our results.

We first discuss some of the most important linguistic areas proposed for the Americas, and then return to the question of how they relate to our study specifically.

## Northwest Coast

This area is represented at its core by Nuu-Chah-Nulth (Wakashan), Halkomelem (Salishan) and more peripherally by Tlingit (Na-Dené) and Haida (Haida) in our sample. Features likely picked up by our questionnaire are the large consonant inventories, including ejectives and uvulars; the small vowel inventories; verb-oriented lexica; VS/VO word order; negation before the verb (in fact, sentence-initial); and numeral classifiers. Most shared features are ancient within involved language families, i.e. they can be traced back within them for their entire accessible history [Thomason 2015]. Their ultimate origin is therefore not clear and may involve language contact, deep genealogical relationships (cf. “Mosan” [Swadesh 1953]), or other aspects of linguistic history too remote to be traceable, a point to which we will return in our general discussion.

## (Northern) California

Northern California has been identified as a linguistic area in the literature, too. It is associated with similar features as the Northwest Coast, including ejective and uvular consonants; numeral classifiers; and –in contradistinction to the Northwest Coast– case marking [Haas 1976; Golla 2011; Haynie 2014]. Given this overlap, Northern California may be considered an extension of the Northwest Coast area [Haynie 2014]. The San Francisco Bay area is the area’s conventionally cited southern limit.

## Mesoamerica

This area is notably visible in our results by the abrupt appearance of green-brownish hues in Mexico that disrupt the more gradient-like transition from light green to bluish green hues. This result is consistent with the view that the Mesoamerican linguistic area has unusually well-defined boundaries [Campbell et al. 1986]. The Mesoamerican linguistic area also stands out by being relatable to a specific, archaeologically visible, and relative to the initial settlement of the continents, late cultural horizon, namely the Olmec culture (ca. 1200–400 BCE) that was foundational to Mesoamerica as a culture area [Campbell et al. 1986].

## Andes and Amazonia

In South America, our cluster analysis reveals a clear tripartite structure, with visibly different structural preferences in the Andes, greater Amazonia (which extends to some extent eastward into and across the Andes, consistent with previous observations [Urban et al. 2019]), and the Southern Cone with Patagonia and Tierra del Fuego. The Andes-Amazonia structure is consistent with a lot of previous literature [Derbyshire 1987, Dixon and Aikhenvald 1999; Van Gijn 2014; Van Gijn and Muysken 2020; Emlen et al. to appear], though recent accounts also here tend to emphasize the gradient nature of spatial structure in typological variation that is not indicative of clear boundaries [Urban 2019, Van Gijn 2014, Emlen et al. to appear]. Features that have been investigated are manifold [Van Gijn 2014, Emlen et al. to appear] and include many that are captured by our questionnaire, including the presence vs. absence of peripheral cases; head-dependent order in phrases; clause-level constituent order; decimal numeral systems, and others.

## The Southern Cone

We also see Patagonia and Tierra del Fuego are clearly set off from both Andes and Amazonia in both our RGB visualization of structural variation in Fig. 1 as well as in cluster analysis. Patagonia is a part of the continent that has been little explored in terms of areal typology, though some shared features have been suggested. These include several that could be picked up by our study: OV syntax [Adelaar with Muysken 2004]; possibly decimal numeral systems [Viegas Barros 2014]; and the rules governing alignment of verbal person marking [Fernández Garay 2012]. A notable feature of some languages that links them to the southern half of the Andes is the occurrence of phonologies that include ejectives and uvulars [Michael and Robertson to appear]. In general, our results are suggestive of strong structural affinities between languages of the Southern Cone.

## Discussion

As is true elsewhere, many of the linguistic areas of the Americas proposed in the literature are “leaky”. This is generally consistent with our results, which show how structural diversity shows a fine spatial structure that tends to change more gradually than abruptly. A clear instance where this is not the case to the same extent is Mesoamerica, which is, however, also consistent with the exceptional nature of this area noted in the literature.

Another property that is true of most of the areas is particularly relevant for our argument: the regional “areal” similarities that have been noted between unrelated languages are often so old that, in fact, they cannot be straightforwardly attributed to known language contact events. In many cases conspicuously shared features reconstruct “up” within known phylogenetic lineages to the respective common ancestor. If any factors other than chance is responsible for the shared feature states, these factors must have already affected these earliest reconstructible ancestors of known phylogenetic lineages, and hence be of considerable antiquity, at least antiquity that transcends what is accessible with the traditional methods of historical linguistics. For instance, the Salishan family of the Pacific Northwest is assumed to have a time depth of 4,000 years, and since relevant diagnostic features of the Northwest Coast linguistic area are shared by Salish languages, these diagnostic features were already in place by the time the common ancestor started to diversify. In this particular case, a distinct possibility is that these features have been inherited within Northwest Coast lineages from a still unknown, older common ancestor, which would push back the time horizon of the features considerably. Indeed, “deep” genealogical connections have been posited within the Northwest Coast (“Mosan”) [Swadesh 1953], a significant portion of Californian languages (“Hokan”, “Penutian”), some Mesoamerican languages [Brown et al. 2011], and some Central Andean languages [Emlen to appear], though the evidence in favor of these groupings has not been sufficient to be universally accepted.

In other cases, such as Amazonia and the Central Andes, perceived similarities are scattered but extremely widespread, so that, at the present state of research, they are too diffuse for a clear contact-based explanation [Payne 1990]. This situation may or may not improve as the social ecologies and Indigenous multilingualism are better understood [Epps 2020], but for now the genesis and also the time depth of these affinities cannot be determined by linguistic means either.

Supplementary material 3: Phylogenetic signals

To quantify the magnitude of phylogenetic signal (i.e., evidence for the horizontal transmission of features within known language phylogenies), we created a similarity matrix for each language family and then calculated the mean distance across all languages. In addition, where we sampled more than two languages per family, we calculated the distance between the pair of languages that are most similar and the standard deviation.

Table 3.1 shows that the mean distance between families ranges from 0.899 for the shallow and close-knit Quechuan family of the Andes to 0.647 for the large and diversified Arawakan family of lowland South America. The onset of diversification of the ancestral language of the Quechuan lineage is dated to approximately 2,000 years BP [Heggarty 2008], while that for Arawakan is assumed to have occurred around 5,000 years BP [Heckenberger 2013]. Mean distances correlate with hypothesized age of the language families very well as families thought to be older show lower values than families that are believed to have formed more recently. This suggests that profiles of phonological and grammatical structure, like the lexicon, accumulate change that can broadly be described as a function of the time since known phylogenies came into existence.

| Family | Standard Deviation | Mean distance | Minimal distance |
| --- | --- | --- | --- |
| Arawakan | NA | 0.647 | 0.647 |
| Chibchan | NA | 0.719 | 0.719 |
| Tupian | NA | 0.736 | 0.736 |
| Athabaskan-Eyak-Tlingit | NA | 0.776 | 0.776 |
| Otomanguean | 0.0894 | 0.781 | 0.679 |
| Uto-Aztecan | 0.0778 | 0.792 | 0.636 |
| Barbacoan | NA | 0.807 | 0.807 |
| Tucanoan | NA | 0.817 | 0.817 |
| Cariban | NA | 0.818 | 0.818 |
| Chicham | NA | 0.865 | 0.865 |
| Miwok-Costanoan | NA | 0.865 | 0.865 |
| Mayan | 0.0210 | 0.870 | 0.847 |
| Tequistlatecan | NA | 0.873 | 0.873 |
| Aymaran | NA | 0.873 | 0.873 |
| Chocoan | NA | 0.879 | 0.879 |
| Quechuan | NA | 0.899 | 0.899 |

Table S3.1. Intra-family mean distance, minimal distance, and where applicable, standard deviation between typological profiles in Indigenous language families of the Americas represented in our sample.

The oldest families, like Arawakan, show a heterogeneous profile and have levels of internal variation that is not appreciably different from the sample as a whole, which we calculate at 0.647. For our set of languages, phylogenetic signals that reflect known families thus likely peter out after around 5,000 years of linguistic diversification, hinting that remaining signals may reflect deeper time affinities as we in fact consider.

# Supplementary material 4: Error estimates

Finally, we evaluated the performance of the error term on the locations where languages are currently spoken; we included this error term because current language locations may be affected by cultural and demographic processes that may be relatively recent and unrelated to early dispersals.

Figure S4.1: Error estimates on when correlating typological distance between languages to their geographical distance from Bering Strait along prehistoric dispersal paths


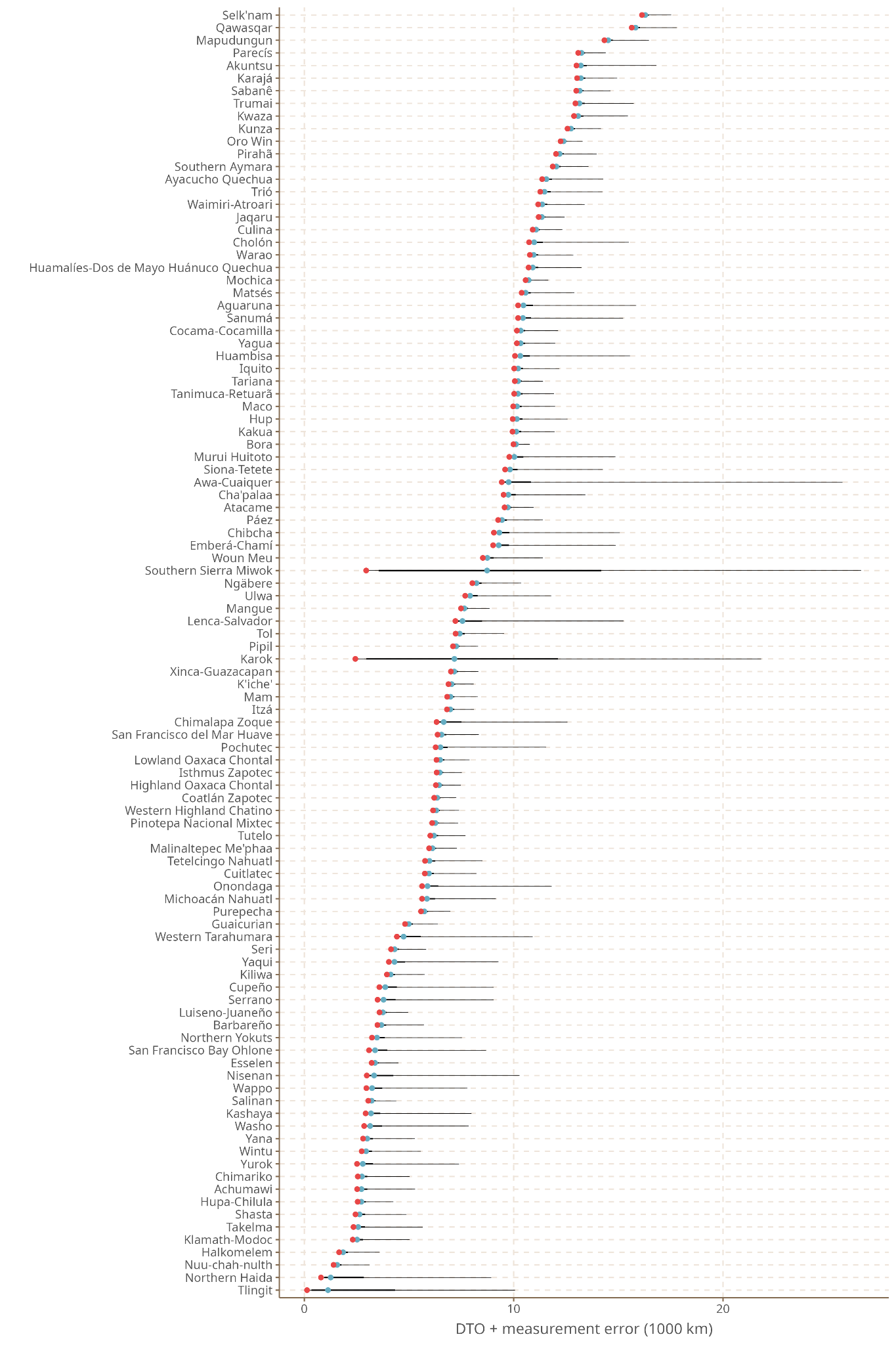


Next, we looked at whether there are any spatial patterns in the measurement error estimation. Figure S4.2 shows (with log scale for easier visualization) that overall, there is no discernible spatial pattern in the mean error estimates. We only observe two large error estimates in North America (we discuss the case of Athabaskan-Eyak-Tlingit briefly in the discussion section of the main text), but the rest of the error estimates are very evenly distributed across the map.


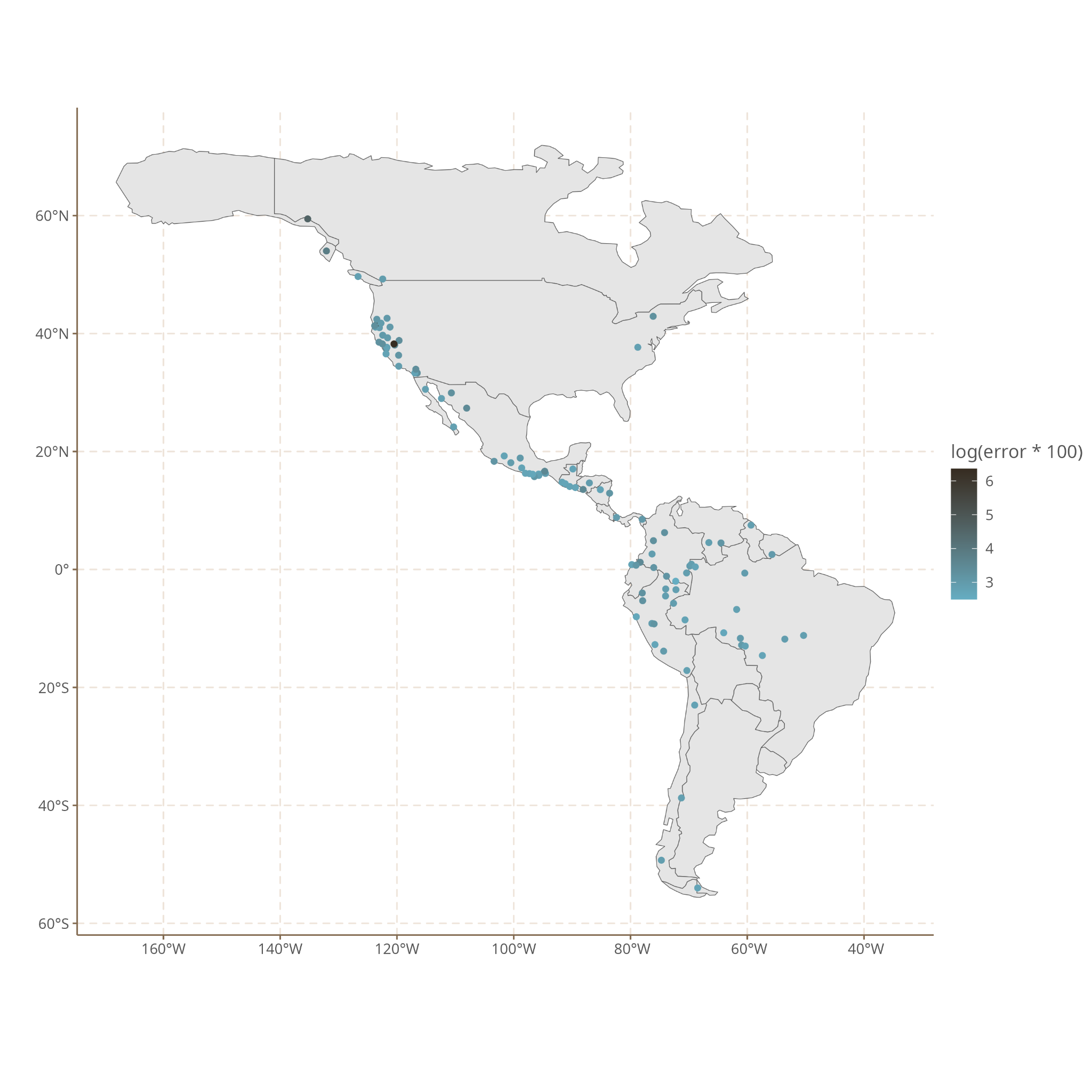


Figure S4.2: Spatial distribution of the mean measurement error of the distance to the origin. This map was made using ggplot2 (Wickham 2016) and sf (Pebesma 2018, 2023).

In terms of distribution across families, we see the same pattern. Table S4.1 shows the mean error estimates by family for those families that are represented by more than one language in our dataset.

| Family | Mean error | N. languages in family |
| --- | --- | --- |
| Arawakan | 0.155 | 2 |
| Athabaskan-Eyak-Tlingit | 0.589 | 2 |
| Aymaran | 0.161 | 2 |
| Barbacoan | 0.278 | 2 |
| Cariban | 0.197 | 2 |
| Chibchan | 0.227 | 2 |
| Chicham | 0.253 | 2 |
| Chocoan | 0.24 | 2 |
| Mayan | 0.156 | 3 |
| Miwok-Costanoan | 3.03 | 2 |
| Otomanguean | 0.153 | 6 |
| Quechuan | 0.198 | 2 |
| Tequistlatecan | 0.162 | 2 |
| Tucanoan | 0.207 | 2 |
| Tupian | 0.195 | 2 |
| Uto-Aztecan | 0.237 | 9 |

Table 4.1: By family mean measurement error of distance to the origin

Consistent with the exploration of error estimates for individual languages, we observe only two larger outliers in the by-family figures, namely Miwok-Costanoan and especially Athabaskan-Eyak-Tlingit (i.e. Na-Dené), which we mention in the discussion section of the main text.

# Supplementary material 5: Marginal effects of the expansion component for all 77 surveyed features

Fig. S5.1 shows the marginal effects of the expansion component for all 77 features we surveyed. Features with mean in red are those for which at least 95% of their posterior distribution does not overlap zero.


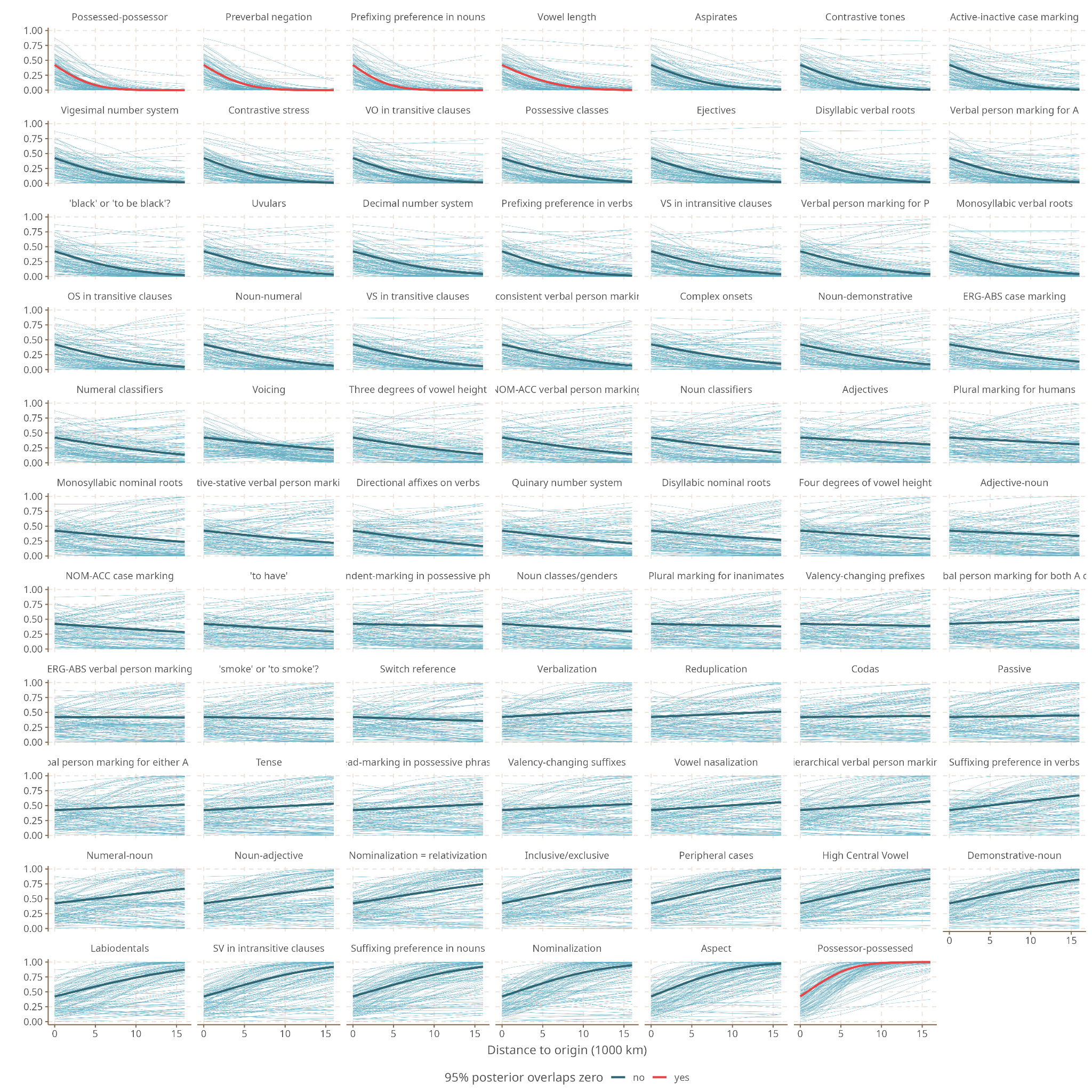


Figure S5.1: Marginal effects of the expansion component for the 77 surveyed features.

Consistent with the impression from the estimates associated with each feature (Fig. 2 in the main text), we observe that effect sizes vary in magnitude and include a large number of features for which the marginal effects are very small.

# Supplementary material 6: Inter-feature correlations


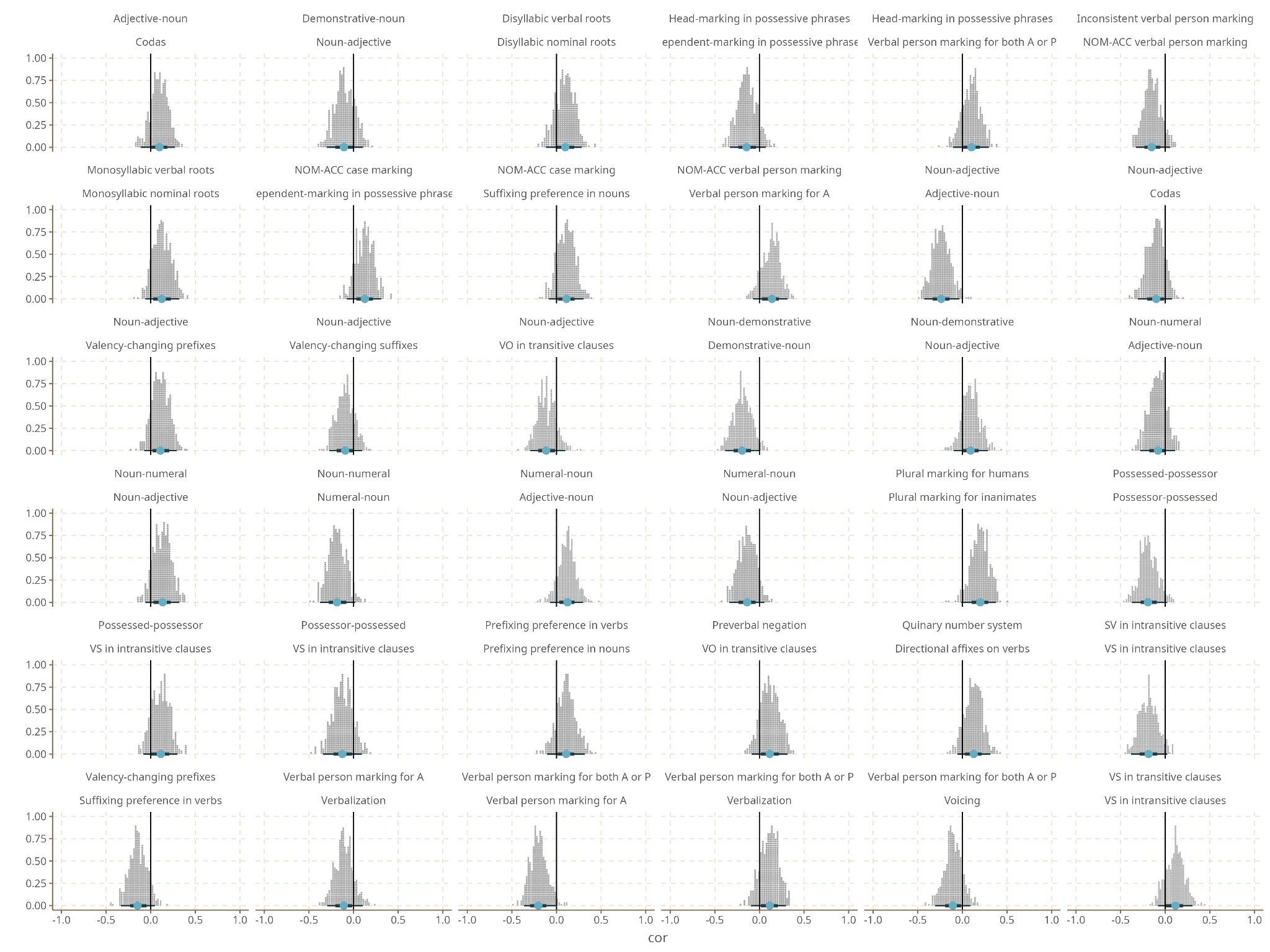


Figure S6.1: Posterior of inter-feature correlations.

Figure S6.1. shows the posterior distribution of some inter-feature correlations once phylogeny and contact are taken into account. Since we have a total of 77 features, showing all correlations would be difficult. Here, we present all correlations with an absolute mean value higher than 0.1. There are 36 such pairs in total. Most posteriors, however, clearly overlap 0 at the 50% and 95% intervals, indicating low certainty about whether the true value is positive or negative. Exceptions concern features that relate to whether, and if so, how, subject and object are referenced on the verb and the order of adjective and noun. These are negatively correlated with each other for our languages (which is not necessarily a general pattern cross-linguistically, Dryer 1988). Other correlations fell below the 0.1 threshold, but can be explored through the *-omega.rds files we provide.

# Supplementary materials 7: supplementary analyses

Our main analysis features several innovations, including a novel probit-based approach to modeling linguistic distributions; the explicit implementation of prehistoric dispersal paths as a component of the model; and the use of a significantly expanded dataset that is tailored specifically to the Americas. Therefore, we wanted to validate our results by comparing them with those obtained using more standard approaches and standard worldwide typological datasets.

## Mixed effect regression controlling for contact and phylogeny

First, we implemented a regression approach. We constructed a Bayesian Mixed Effect regression model that included group-level effects controlling for known language phylogenies (using, like in our main analysis, the standard Glottolog phylogenetic classification of world’s languages [Hammarström et al. 2023). For control of areal effects, we used Autotyp areas, whose delimitation is “informed by current knowledge of the historical, genetic, anthropological, and archeological record” [Nichols et al. 2013]

We included the distance of a language from the point of entry of humans into the Americas along inferred dispersal paths as predictor into the model. Our response was the typological profile of that language, expressed as a single figure obtained by MCA, a variant of Principal Component Analysis [Le Roux and Rouanet 2010].

We built two models: For the first we used our own tailormade dataset. For the second model, we used data from Grambank [Skirgard et al 2023], a database of typological structures with worldwide coverage. While there is some overlap, the Grambank dataset looks at many different features, which, unlike our principal dataset, are not attuned to the specific loci of variability in the Americas.

Both models reveal a clear trend for typological distance to increase with greater distance of languages along prehistoric dispersal paths, but also large uncertainty in the estimates (Figures S7.1 and S 7.2). This experiment is thus inconclusive, but it does not disagree with our results.


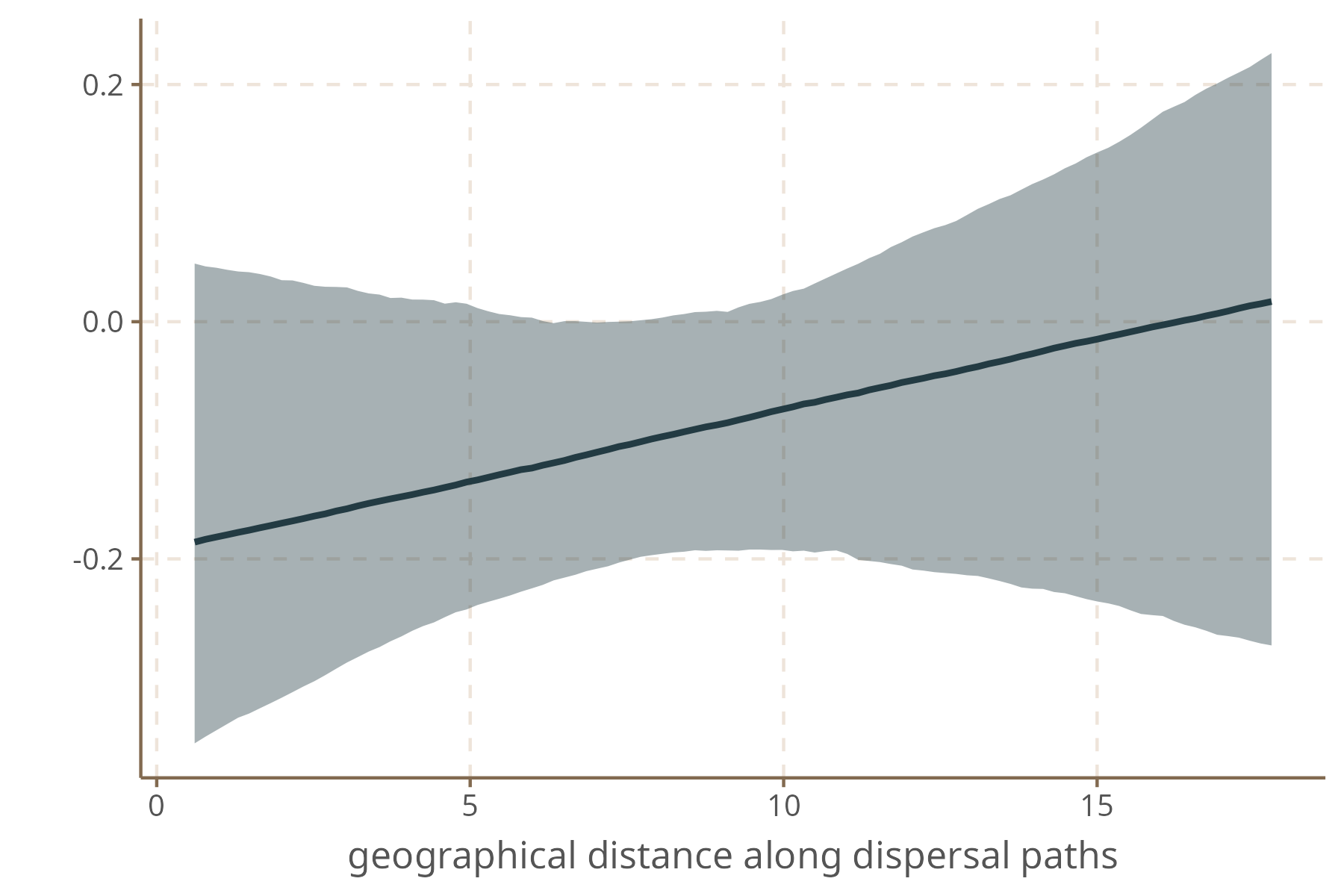


Figure S7.1: Regression model predicting typological profile from distance to the origin with Grambank data.


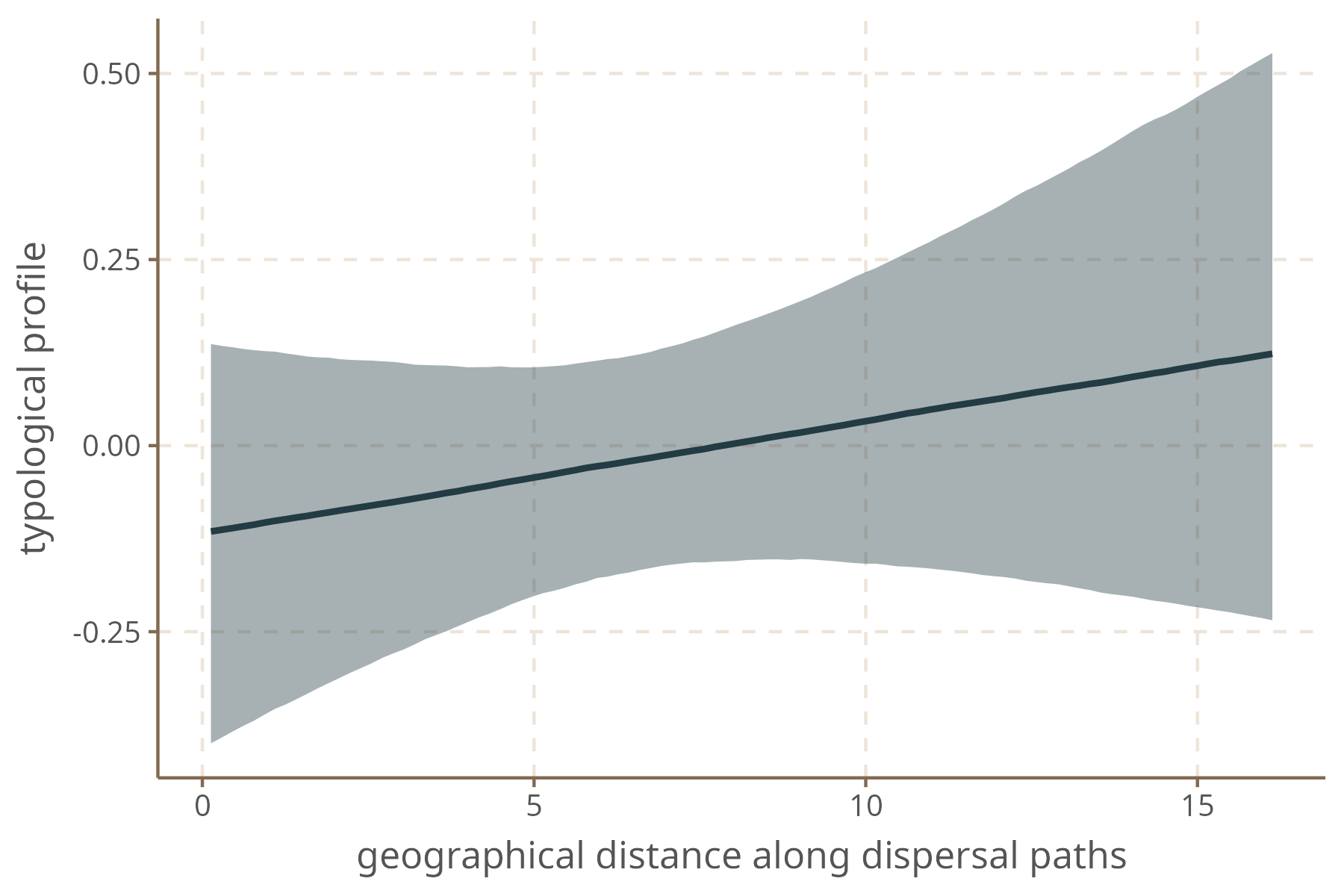


Figure S7.2: Regression model predicting typological profile from the distance to the origin with our data

## Geographical axis effects in the Americas and in Eurasia with fictive points of entry

We further wanted to test whether our Americas-specific interpretation of gradient structure in typological variation can also be observed elsewhere, especially given that large-scale comparative research now shows similarities between languages across large, including continent sized, areas [Dryer 1989, Bickel 2020]. To investigate this, we used Grambank and WALS data for the Eurasian continent. To express the geographical position of each Eurasian language, we considered a west-to-east geographical axis starting in Iceland and reaching Japan. For comparability also in terms of interpretation, this can be thought of as assuming a fictive point of entry into Eurasia from Iceland with following eastward dispersal. Since this is not the way humans actually colonized Eurasia in prehistory, we expected that we would not see spatial structure in the phonological and grammatical profiles in a way that is comparable to the Americas. Like in our regression analyses for the Americas, we expressed structural profiles as a single variable using dimensionality reduction via MCA. To control for phylogeny and, to a degree, contact, in this case we sampled a random language from each family. We then tried to predict the MCA value derived from the languages’ structural profile from the fictive distance to Iceland in a simple linear model. We repeated this process 1,000 times.

Our rationale for implementing this approach is based on the following points: First, it is a much less conservative approach than the model we present in the main text. The controls are less strict, and thus, the chances of seeing an effect in the Eurasian datasets is much higher, giving the data the best chance to show that our main results might be spurious. The second is technical. We wanted to see to what extent the type of result we see in our main analysis can be replicated using a drastically different technique.

Finally, we wanted to explore further our result that only a relatively small subset of features seem to show somewhat stronger gradient distributions in the America. For that reason we build two different simulations with our data. In the first one, we use all features to perform the MCA, while in the second we only use those features which are most stable according to a meta-analysis based on seven studies [Dediu and Cysow 2013].

We fitted a total of ten simulations, looking at the Americas and Eurasia with the full Grambank and WALS data, and with subsets that only include stable features. In addition, we looked at the Americas on the basis of our own full tailormade dataset, and a subset that again only includes stable features.


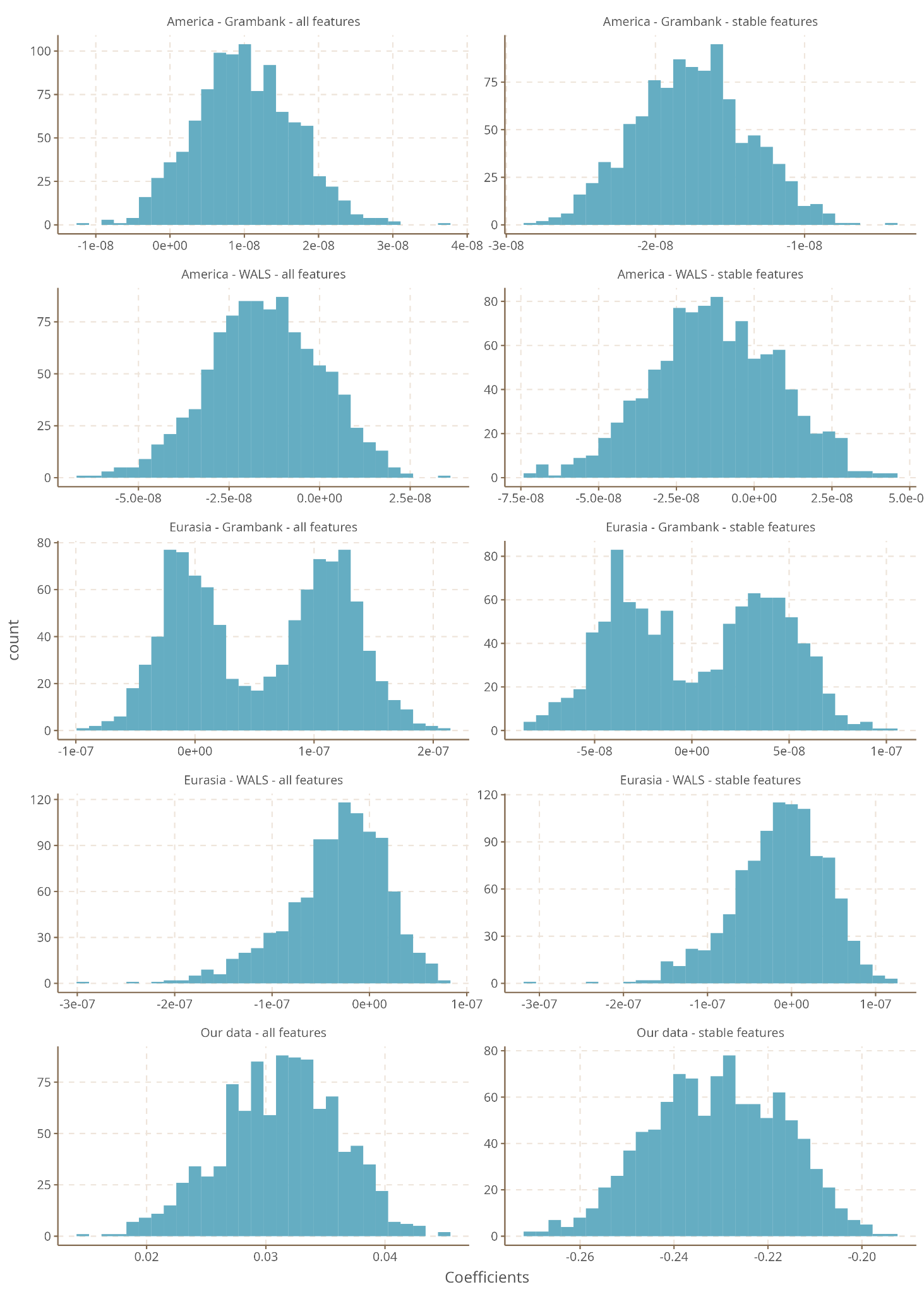


Figure S7.3: coefficients of the family-sampling simulations. The sign of the coefficient does not matter, but the effect size does.

Figure S7.3 shows the results of all eight simulations. The effect sizes of the Grambank and WALS datasets are much smaller than the effect sizes we observe for our dataset, which has been designed to include properties known to be variable n languages of the Americas; also, the effect size for the simulation using more stable features produces a disproportionately stronger effect sizes in our dataset. These results confirm our observation that this group of features is more likely to reflect a historical signal related to American prehistory.

We tend to interpret the small effect sizes obtained on the basis of the WALS and Grambank datasets vis-a-vis our American dataset against the background of the results of our model, which showed that a small subset of features contain a gradient signal consistent with prehistoric expansion paths. In a regression approach based on a single, aggregated typological profile, these effects will likely be masked by the large number of uninformative features, to produce the negligible effect sizes of latitudinal and longitudinal geographical axes at large. This is in line with recent work on the geographical distribution of features, which show that in other historical scenarios that involve demographic expansion, such as the relatively recent dispersal of Polynesian languages in remote Oceania [Pawley and Green 1973, Green 1981, Irwin 1994, Kirch and Green 2001], typical phylogenetic and areal controls included in relevant models control for confounds insufficiently, that attention needs to be paid to the selection of features, and that historical expansion events need to be taken into account carefully. Our results are consistent with this conclusion, and suggest that they are relevant also for events that took place in more remote prehistory.

# Supplementary material 8: by-feature balanced accuracy

Figure 8.1 shows the by-feature balanced accuracy all three MultivAreate probit models we built.


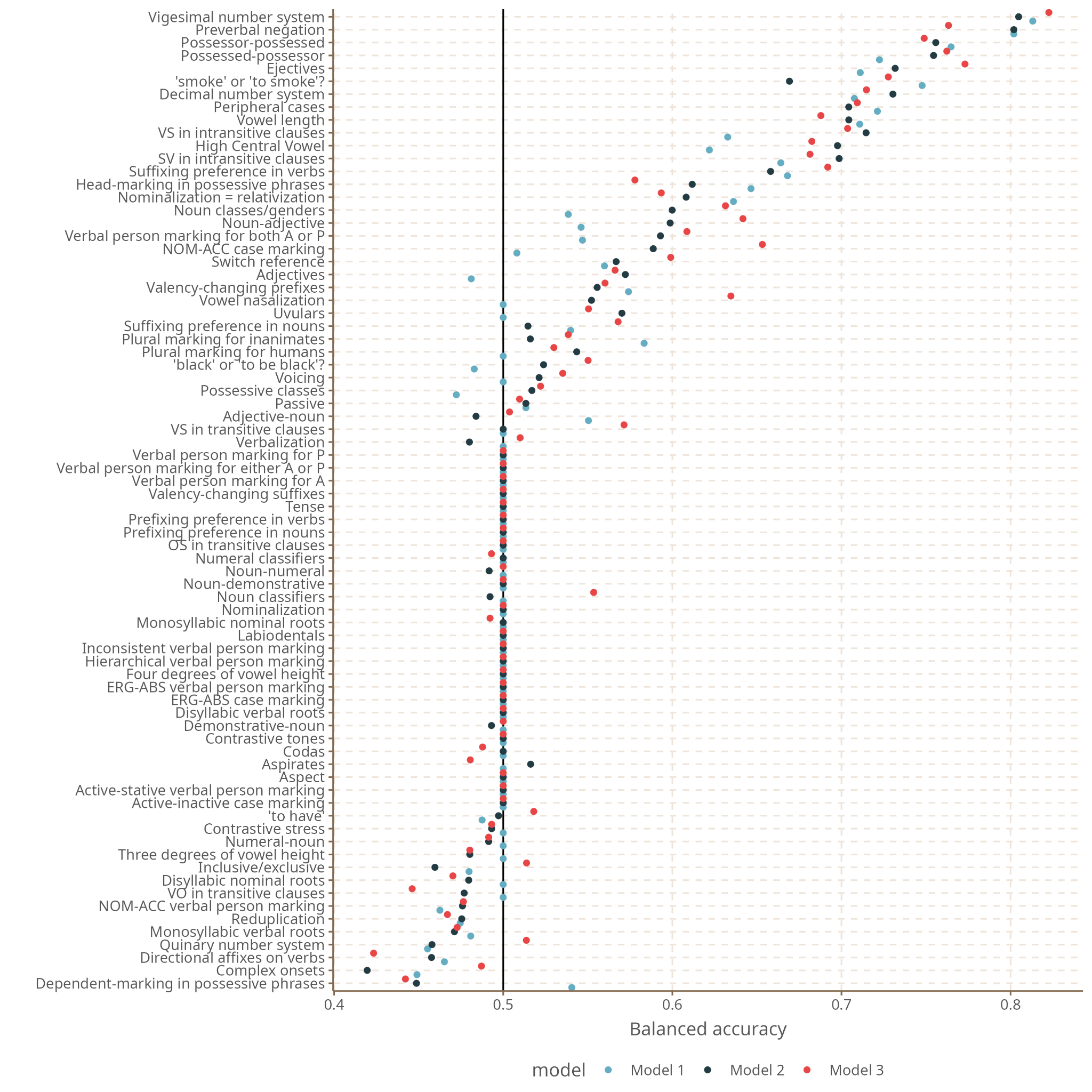


Figure 8.1: By-feature balanced accuracy for Model 1, Model 2 and Model 3.

Notably, there are many features which exhibited essentially no spatial patterning, and because of this, the models performed no better than chance in trying to predict their values (balanced accuracy <= 0.5); these features are those in the middle of the plot in Fig. 8.1. In contrast, features for which the models achieved relatively high accuracy are features which are strongly clustered in geographical space. Excluding all features for which all models perform at chance level or worse, Model 1 achieved the highest balanced accuracy for 12 features, Model 2 for 10, and Model 3 performed best, yielding the highest balanced accuracy for 20 features.

While taken for the whole set of features all three models performed very similarly, we do see a small but clear impact of the expansion effects on a handful of features as Models 2 and 3 outperformed Model 1, showing that the expansion component helps predict feature values. At the same time, Model 3 outperformed Model 2 with regard to multiple features, sometimes by a large margin. At least for a subset of our features, adding uncertainty about the actual demographic history of the people speaking individual languages, possibly including later migration or cultural events affecting language use and distribution, helps the Model predict feature values better.

Supplementary material 9: sampled languages and consulted sources

| Language | Family | Glottocode | ISO 639-3 code | Latitude | Longitude | Sources |
| --- | --- | --- | --- | --- | --- | --- |
| Achumawi | Palaihnihan | achu1247 | acv | 41.10 | -121.19 | De Angulo and Freeland 1930, Nevin 1998 |
| Aguaruna | Chicham | agua1253 | agr | -5.30 | -77.92 | Wipio Deicat 1996, Overall 2007 |
| Akuntsu | Tupian | akun1241 | aqz | -12.83 | -60.97 | Aragon 2014 |
| Atacame | Atacame | atac1235 |  | 0.83 | -79.78 | Seler 1902, Constenla Umaña 1991, Adelaar with Muysken 2004, Adelaar 2005 |
| Awa-Cuaiquer | Barbacoan | awac1239 | kwi | 1.22 | -78.34 | Curnow 1997, Calvache Dueñas 2000 |
| Ayacucho Quechua | Quechuan | ayac1239 | quy | -13.85 | -74.32 | Parker 1969 |
| Bora | Boran | bora1263 | boa | -2.00 | -72.26 | Thiesen and Weber 2012 |
| Cha'palaa | Barbacoan | caya1244 |  | 0.71 | -79.05 | Abrahamson 1962, Lindskoog and Brend 1962, Lindskoog and Lindskoog 1964, Vittadello 1988, Wiebe and Wiebe 2007 |
| Chibcha | Chibchan | chib1270 | chb | 6.25 | -74.17 | Lugo [1619]1978, Constenla Umaña 1981, Anonymous 1987, Ostler 1993, 1994, Adelaar 1995, Adelaar with Muysken 2004 |
| Chimalapa Zoque | Mixe-Zoque | chim1300 | zoh | 16.65 | -94.69 | Johnson 2000 |
| Chimariko | Chimariko | chim1301 | cid | 41.00 | -123.00 | Jany 2009 |
| Cholón | Hibito-Cholón | chol1284 | cht | -9.23 | -75.96 | Alexander-Bakkerus 2005 |
| Coatlán-Loxicha Zapotec | Otomanguean | coat1243 | zps/zpx | 16.15 | -96.77 | Beam de Azcona 2004 |
| Cocama-Cocamilla | Tupian | coca1259 | cod | -4.50 | -74.00 | Vallejos 2016 |
| Cuitlatec | Cuitlatec | cuit1236 | cuy | 18.10 | -100.50 | Hendrichs 1939, McQuown 1941, Escalante 1962, Valiñas Coalla et al. 1984, Colville 1986 |
| Culina | Arawan | culi1244 | cul | -8.55 | -70.66 | Dienst |
| Emberá-Chamí | Chocoan | embe1262 | cmi | 4.88 | -76.07 | Aguirre Licht 1999 |
| Esselen | Esselen | esse1238 | esq | 36.55 | -121.87 | Shaul 1995 |
| Halkomelem | Salishan | halk1245 | hur | 49.25 | -122.44 | Suttles 2004 |
| Highland Oaxaca Chontal | Tequistlatecn | high1242 | chd | 16.18 | -95.73 | Turner 1966 |
| Huamalíes-Dos de Mayo Huánuco Quechua | Quechuan | huam1248 | qvh | -9.16 | -76.34 | Weber 1989, Weber et al. 1998 |
| Huambisa | Chicham | huam1247 | hub | -3.99 | -77.98 | Peña 2015 |
| Hupa-Chilula | Athabaskan-Eyak-Tlingit | hupa1239 | hup | 41.10 | -123.68 | Goddard 1905, Golla 1970, 1996, Sapir 2001 |
| Hup | Naduhup | hupd1244 | jup | 0.59 | -69.84 | Epps 2008 |
| Iquito | Zaparoan | iqui1243 | iqu | -3.31 | -73.97 | Brown 2004, Lai 2009 |
| Isthmus Zapotec | Otomanguean | isth1244 | zai | 16.45 | -94.84 | Pickett 2007, Pickett et al. 1998, Pickett et al. 2010 |
| Itzá | Mayan | itza1241 | itz | 17.03 | -89.87 | Hofling with Tesucún 1997, 2000 |
| Jaqaru | Aymaran | jaqa1244 | jqr | -12.74 | -75.81 | Hardman 2000 |
| Kakua | Kakua-Nukak | cacu1241 | cbv | 0.89 | -69.57 | Bolaños 2016 |
| Karajá | Nuclear-Macro-Je | kara1500 | kpj | -11.19 | -50.35 | Ribeiro 2012 |
| Karuk | Karuk | karo1304 | kyh | 41.82 | -123.31 | Bright 1957 |
| K’iche‘ | Mayan | kich1262 | quc | 14.53 | -91.17 | Colville 1986, Larsen 1988, Ixchajchal Bath et al. 1996 |
| Kiliwa | Cochimi-Yuman | kili1268 | klb | 30.55 | -115.12 | Mixco 1971, 1985, 2000 |
| Kunza | Kunza | kunz1244 | kuz | -23.00 | -69.00 | Sáez Godoy et al. 1974, Adelaar with Muysken 2004, Peyró Garcia 2005 |
| Kwaza | Kwaza | kwaz1243 | xwa | -11.67 | -61.18 | Van der Voort 2004 |
| Lenca-Salvador | Lencan | lenc1243 | len | 13.56 | -88.11 | Lehmann 1920, Campbell 1976a, Constenla Umaña 1991, Del Río Urrutia 1999 |
| Lowland Oaxaca Chontal | Tequistlatecan | lowl1260 | clo | 15.95 | -95.69 | Waterhouse 1962, 1985, O’Connor 2007, O’Connor and Kroefges 2008, Maddieson et al. 2009 |
| Maco | Jodi-Saliban | maco1239 | wpc | 4.56 | -66.58 | Rosés Labrada 2015 |
| Malinaltepec Me'phaa | Otomanguean | mali1285 | tcf | 17.21 | -98.63 | Suárez 1983, Wichmann 2007, 2009 |
| Mam | Mayan | mann1241 | mam | 14.80 | -91.72 | England 1983, Pérez Alonso et al. n.d. |
| Mangue | Otomanguean | moni1237 | mom | 13.53 | -85.17 | Lehmann 1920, Quirós Rodríguez 1985 |
| Mapudungun | Araucanian | mapu1245 | arn | -38.74 | -71.28 | Augusta [1916]1996, Smeets 2008, Sadowsky et al. 2013 |
| Matsés | Pano-Tacanan | mats1244 | mcf | -5.74 | -72.63 | Fleck 2003 |
| Michoacán Nahuatl | Uto-Aztecan | mich1245 | ncl | 18.34 | -103.36 | Sischo 1979 |
| Mochica | Mochica | moch1259 | omc | -8.02 | -79.00 | Cerrón-Palomino 1995, Salas García 2002, Adelaar with Muysken 2004, Hovdhaugen 2004 |
| Murui Huitoto | Huitotoan | muru1274 | huu | -1.14 | -73.83 | Wojtylak 2017 |
| Ngäbere | Chibchan | ngab1239 | gym | 8.83 | -82.39 | Alphonse 1956, Quesada Pacheco 2008 |
| Nisenan | Maiduan | nise1244 | nsz | 38.05 | -120.39 | Eatough 1999 |
| Northern Haida | Haida | nort2938 | hdn | 54.00 | -132.08 | Enrico 2003 |
| Northern Yokuts | Yokutsan | yoku1256 | yok | 36.32 | -119.72 | Newman 1944, Collord 1968, Weigel 2005 |
| Nuu-chah-nulth | Wakashan | nuuc1236 | nuj | 49.67 | -126.67 | Davidson 2002 |
| Onondaga | Iroquoian | onon1246 | ono | 42.92 | -76.12 | Barrie 2015, Woodbury 2018 |
| Oro Win | Chapacuran | orow1243 | orw | -10.74 | -64.03 | Popky 1999 |
| Páez | Páez | paez1247 | pbb | 2.62 | -76.31 | Castillo i Orosco and Uricoechea [1877]1968, Slocum and Gerdel 1983, Jung 2008 |
| Parecís | Arawakan | pare1272 | pab | -14.59 | -57.41 | Barros Brandão 2014 |
| Pinotepa Nacional Mixtec | Otomanguean | pino1237 | mio | 16.30 | -93.00 | Bradley 1970 |
| Pipil | Uto-Aztecan | pipi1250 | ppl | 13.89 | -89.44 | Campbell 1985, Colville 1986 |
| Pirahã | Pirahã | pira1253 | myp | -6.78 | -61.82 | Everett 1986 |
| Pochutec | Uto-Aztecan | poch1244 | xpo | 15.74 | -96.47 | Boas 1917, Bartholomew 1980 |
| Purepecha | Tarascan | pure1242 | tsz | 19.25 | -101.63 | LeCron Foster 1969, Campbell et al. 1986, Chamoreau 2000 |
| Qawasqar | Kawesqar | qawa1238 | alc | -49.30 | -74.71 | Clairis 1985, Aguilera 2001 |
| Sabanê | Nambiquaran | saba1268 | sae | -12.99 | -60.34 | Antunes de Araujo 2004 |
| San Francisco del Mar Huave | Huavean | sanf1258 | hue | 16.28 | -94.57 | Kim 2008 |
| Sanumá | Yanomamic | sanu1240 | xsu | 4.50 | -64.50 | Borgman, Wilbert 1962 |
| Selk‘nam | Chonan | onaa1245 | ona | -54.00 | -68.50 | Ocampo 1982, Rojas Berscia 2014 |
| Seri | Seri | seri1257 | sei | 28.99 | -112.38 | Marlett 1981, Moser and Marlett 1994, 1995, 2004 |
| Serrano | Uto-Aztecan | serr1255 | ser | 33.95 | -116.77 | Anderton 1988. |
| Shasta | Shastan | shas1239 | sht | 41.76 | -122.74 | Silver 1966, Bright and Olmsted 1959 |
| Siona-Tetete | Tucanoan | sion1247 | snn | 0.32 | -76.02 | Wheeler 1970, Bruil 2014 |
| Southern Aymara | Aymaran | sout2996 | ayc | -17.14 | -70.34 | Coler 2014 |
| Southern Sierra Miwok | Miwok-Costanoan | sout2985 | skd | 38.24 | -120.51 | Broadbent 1964 |
| Takelma | Takelma | take1257 | tkm | 42.42 | -123.45 | Kendall 1977, Sapir 1990, Golla 2011 |
| Tanimuca-Retuarã | Tucanoan | tani1257 | tnc | -0.59 | -70.39 | Strom 1992 |
| Tariana | Arawakan | tari1256 | tae | 0.44 | -68.90 | Aikhenvald 2003 |
| Tetelcingo Nahuatl | Uto-Aztecan | tete1251 | nhg | 18.89 | -98.89 | Tuggy 1979 |
| Tlingit | Athabaskan-Eyak-Tlingit | tlin1245 | tli | 59.44 | -135.29 | Naish 1979,  Leer 1991  Crippen 2012, Eggleston 2013, Twitchell 2016 |
| Tol |  |  | jic |  |  | Dennis and Royce de Dennis 1983, Holt 1999 |
| Trió | Cariban | trio1238 | tri | 2.54 | -55.76 | Carlin 2004 |
| Trumai | Trumai | trum1247 | tpy | -11.81 | -53.57 | Guirardello 1999 |
| Tutelo | Siouan | tute1247 | tta | 37.67 | -78.75 | Oliverio 1996 |
| Ulwa | Misumalpan | ulwa1239 | ulw | 12.93 | -83.58 | Green 1999 |
| Waikuri |  |  |  |  |  | Zamponi 2004 |
| Waimiri-Atroari | Cariban | waim1253 | atr | -0.62 | -60.43 | Bruno 2003 |
| Wappo | Yuki-Wappo | wapp1239 | wao | 38.25 | -122.50 | Radin 1929, Thompson et al. 2006 |
| Warao | Warao | wara1303 | wba | 7.51 | -59.35 | Romero-Figeroa 1997 |
| Washo | Washo | wash1253 | was | 38.82 | -119.64 | Kroeber 1907, Jacobsen 1964, 1990 |
| Western Highland Chatino | Otomanguean | west2644 | ctp | 16.25 | -97.32 | Rasch 2002 |
| Western Tarahumara | Uto-Aztecan | lowl1254 | tac | 27.35 | -108.06 | Burgess 1984 |
| Wintu | Wintuan | wint1259 | wit | 39.72 | -122.44 | Pitkin 1984, 1985 |
| Woun Meu | Chocoan | woun1238 | noa | 8.50 | -78.00 | Loewen 1954, Holmer 1963, Mejía Fonnegra 2000, Aguirre Licht 2009 |
| Xinca-Guazacapan | Xincan | xinc1246 | xin | 14.07 | -90.42 | Sachse 2010 |
| Yagua | Peba-Yagua | yagu1244 | yad | -3.43 | -72.22 | Payne 1985, Payne and Payne 1990 |
| Yana | Yana | yana1271 | ynn | 39.28 | -121.61 | Sapir 1922, 1923, Sapir and Swadesh 1960 |
| Yaqui | Uto-Aztecan | yaqu1251 | yaq | 29.94 | -110.68 | Dedrick and Casad 1999 |
| Yurok | Algic | yuro1248 | yur | 41.33 | -123.82 | Robins 1958, Garrett 2014, Garrett et al. 2005 |

Abrahamson, Arne. 1962. Cayapa: grammatical notes and texts. In: Elson (ed.), 217-247.

Adelaar, Willem. 1995. Les catégories verbales ‘conjugaison’ et ‘genre’ dans les grammaires de lalangue chibcha. Amerindia 19/20: 173-182.

Adelaar, Willem F.H. 2005. El esmeraldeño – un idioma de la costa del Ecuador. En: Sabine

Dedenbach-Salazar Sáenz (ed.): Contribuciones a las lenguas y culturas de los Andes. Homenaje a Alfredo Torero, 233-246. Aachen: Shaker.

Adelaar, Willem F.H., with the collaboration of Pieter C. Muysken. 2004. The Languages of the Andes.Cambridge: Cambridge University Press.

Aguilera F., Oscar E. 2001. Gramática de la lengua kawésqar. Temuco: Corporación Nacional de Desarollo Indígena.

Aguirre Licht, Daniel. 1999. Embera. Munich: Lincom Europa.

Aguirre Licht, D. 2009. Choco languages. In: Keith Brown and Sarah Ogilvie (eds.): Conciseencyclopedia of languages of the world, 224-238. Oxford: Elsevier.

Aikhenvald, Alexandra Y. 2003. A grammar of Tariana, from northwest Amazonia. Cambridge: Cambridge University Press.

Alexander-Bakkerus, Astrid. 2005. Eighteenth-century Cholón. Utrecht: LOT.

Alphonse, Ephraim S. 1956. Guaymí grammar and dictionary with some ethnological notes. Washington: United States Government Printing Office.

Anderton, Alice Jeanne. 1988. The language of the Kitanemuks of California. Dissertation, University of California Los Angeles.

de Angulo, Jaime, and Lucy S. Freeland. 1930. The Achumawi language. International Journal of American Linguistics 6: 77-120.

Anonymous. 1987. ‘Diccionario y gramática chibcha’. Manuscrito anónimo de la

Biblioteca Nacional de Colombia. Ed. By González de Pérez, María Stella. Bogotá: Instituto Caro y Cuervo.

Antunes de Araujo, Gabriel. 2004. A grammar of Sabanê: a Nambikwaran language. Utrecht: LOT.

Aragon, Carolina Coelho. 2014. A grammar of Akuntsú, a Tupían language. Dissertation, University of Hawai'i at Mānoa.

Augusta, Felix José de. [1916] 1996. Diccionario araucano-español y español-araucano, vol. 1: araucano-español. Santiago de Chile: Ediciones Cerro Manquehue.

Barrie, Michael. 2015. A grammar of Onondaga. München: LINCOM Europa.

Barros Brandão, Ana Paula. 2014. A reference grammar of Paresi-Haliti (Arawak). Dissertation, University of Texas at Austin.

Bartholomew, Doris. 1980. Otomanguean influence on Pochutla Aztec. International Journal of American Linguistics 46 (2): 106-116.

Beam de Azcona, Rosemary Grace. 2004. A Coatlán-Loxicha Zapotec gramar (Mexico). Dissertation,University of California, Berkeley.

Beck, David. 2000. Grammatical convergence and the genesis of diversity in the Northwest Coast *Sprachbund*. Anthropological Linguistics 42 (2): 147-213.

Bolaños, Katherine. 2016. A grammar of Kakua. Utrecht: LOT.

Boas, Franz. 1917. El dialecto mexicano de Pochutla, Oaxaca. International Journal of American Linguistics 1 (1): 9-44.

Borgman, Donald M. 1990. Sanuma. In: Desmond C. Derbyshire and Geoffrey K. Pullum (eds.): Handbook of Amazonian Languages, vol. 2, 15-248. Berlin/New York/Amsterdam: Mouton de Gruyter.

Bradley, C. Henry. 1970. A linguistic sketch of Jicaltepec Mixtec. Norman: Summer Institute of Linguistics of the University of Oklahoma.

Bright, William. 1957. The Karok language. Berkeley/Los Angeles: University of California Press.

Bright, William, and David L. Olmsted. 1959. A Shasta Vocabulary. Kroeber Anthropological Society Papers 20: 1-55.

Broadbent, Sylvia M. 1964. The Southern Sierra Miwok language. Berkeley/Los Angeles: University of California Press.

Brown, Marc C. 2004. Topics in Iquito syntax: word order, possession, and nominal discontinuity. MA thesis, University of Texas at Austin.

Bruil, Martine. 2014. Clause-typing and evidentiality in Ecuadorian Siona. Dissertation, Leiden University.

Bruno, Ana Carla. 2003. Waimiri Atroari grammar: some phonological, morphological, and syntactic aspects. Dissertation, University of Arizona.

Burgess, Don. 1984. Western Tarahumara. In: Ronald W. Langacker (ed.): Studies in Uto-Aztecan grammar. Vol. 4: Southern Uto-Aztecan grammatical sketches, 2-149. Dallas: Summer Institute of Linguistics/The University of Texas at Arlington.

Calvache Dueñas, Rocío. 2000. Fonología y aproximación a la morfosintaxis del Awa Pit. In:

González de Pérez and Rodríguez de Montes (eds.), 97-116.

Campbell, Lyle. 1976a. The last Lenca. International Journal of American Linguistics 42 (1): 73-78.

Campbell, Lyle. 1985. The Pipil language of El Salvador. Berlin/New York/Amsterdam: Mouton.

Campbell, Lyle, Terrence Kaufman, and Thomas C. Smith-Stark. 1986. Mesoamerica as a linguistic area. Language 62(3): 530-570.

Carlin, Eithne B. 2004. A grammar of Trio: a cariban language of Suriname. Frankfurt am Main: Peter Lang.

Castillo i Orosco, Eujenio del, and Ezequiel Uricoechea. [1877]1968. Vocabulario Páez-Castellano, catecismo, nociones gramaticales i dos pláticas, conforme a lo que escribió el señor Eujenio del Castillo i Orosco, Cura de Tálaga, con adiciones, correcciones i un vocabulario castellano-paez por Ezequiel Uricoechea. Nendeln/Liechtenstein: Klaus reprint.

Cerrón-Palomino, Rodolfo. 1995. La lengua de Naimlap (reconstrucción y obsolescencia del mochica). Lima: Fondo Editorial de la Pontificia Universidad Católica del Perú.

Chamoreau, Claudine. To appear. Purepecha: an isolate non-Mesoamerican language in

Mesoamerica. In: Søren Wichmann (ed.): The Languages of Middle America: a Comprehensive Guide. Berlin: Mouton de Gruyter.

Clairis, Christos. 1985. El Qawasqar. Lingüística fueguina, teoría y descripción. Valdivia:

Universidad Austral de Chile, Facultad de Filosofía y Humanidades,

Coler, Matt. 2014. A grammar of Muylaq' Aymara: Aymara as spoken in Southern Peru. Leiden: Brill. Leiden/Boston: Brill.

Collord, Thomas L. 1968. Yokuts grammar: Chukchansi. Dissertation, University of California at Berkeley.

Colville, Jeffrey Keith. 1986. The structure of Mesoamerican numeral systems with a comparison to non-mesoamerican systems. Dissertation, Tulane University.

Constenla, Adolfo. 1981. Comparative Chibchan Phonology. Dissertation, University ofPennsylvania.

Constenla Umaña, Adolfo. 1991. Las lenguas del area intermedia: introducción a su estudio areal. San José: Editorial de la Universidad de Costa Rica.

Crippen, James. 2012. The basics of Tlingit verbal structure. Ms. 379pp.

Curnow, Timothy Jowan. 1997. A grammar of Awa Pit (Cuaiquer): an indigenous language of southwestern Colombia. Dissertation, Australian National University.

Davidson, Matthew. 2002. Studies in Wakashan (Nootkan) grammar. Dissertation, State University of New York at Buffalo.

Dedrick, John M., and Eugene H. Casad. 1999. Sonora Yaqui language structures. Tucson:University of Arizona Press.

Del Río Urrutia, Ximena. 1999. El Lenca de Chilanga. Filología y Lingüística 25 (1): 193-209.

Dennis, Ronald K., and Margaret Royce de Dennis. 1983. Diccionario Tol (Jícaque)-Español y Español-Tol (Jícaque). Tegucigalpa: Instituto Lingüístico de Verano/Instituto Hondureño de Antropología e Historia.

Dienst, Stefan. 2014. A grammar of Kulina. Berlin/Boston: Walter De Gruyter.

Eatough, Andrew. 1999. Central Hill Nisenan texts with grammatical sketch. Berkeley /Los Angeles: University of California Press.

Eggleston, Keri M. 2013. 575 Tlingit verbs: a study of Tlingit verb paradigms. Dissertation, University of Alaska Fairbanks.

England, Nora C. 1983. A grammar of Mam, a Mayan language. Austin: University of Texas Press.

Enrico, John. 2003. Haida syntax. Lincoln: University of Nebraska Press.

Epps, Patience. 2008. A grammar of Hup. Berlin, Mouton de Gruyter.

Escalante Hernández, Roberto. 1962. El Cuitlateco. México: Instituto Nacional de Antropología e Historia.

Evans, Nicholas. 2018. Linguistic divergence under contact. In: Michela Cennamo and Claudia Fabrizio (eds.): Historical Linguistics 2015: Selected papers from the 22nd International Conference on Historical Linguistics, Naples, 27-31 July 2015, 563-592. Amsterdam/Philadelphia: John Benjamins.

Everett, Daniel L. 1986. Pirahã. In :Desmond C. Derbyshire and Geoffrey K. Pullum (eds.): Handbook of Amazonian languages, vol.1, 200-325. Berlin/New York/Amsterdam: Mouton de Gruyter.

Fleck, David W. 2003. A grammar of Matses. Dissertation, Rice University.^

Garrett, Andrew. 2014. Basic Yurok grammar. Berkeley: University of California at Berkeley.

Garrett, Andrew, Juliette Blevins, and Lisa Conathan. 2005. Preliminary Yurok dictionary. Berkeley: University of California at Berkeley.

Goddard, P.E. 1905. The morphology of the Hupa language. Berkeley: The University Press.

Golla, Victor K. 1970. Hupa grammar. Dissertation, University of California at Berkeley.

Golla, Victor K. 1996. Hupa language dictionary. Hoopa: Hoopa Valley Tribal Council.

Golla, Victor. 2011. California Indian languages. Berkeley/Los Angeles/London: University of California Press.

Green, Thomas M. 1999. A lexicographic study of Ulwa. Dissertation, Massachusetts Institute of Technology.

Guirardello, Raquel 1999. A reference grammar of Trumai. Dissertation, Rice University.

Hardman, M.J. 2000. Jaqaru. Munich: Lincom Europa.

Hendrichs, P. R. 1939. Un estudio preliminar sobre la lengua cuitlateca de San Miguel Totolapan, Gro. El México Antiguo 4: 329-362.

Hofling, Charles Andrew, with Félix Fernando Tesucún. 1997. Itzaj Maya-Spanish-English dictionary = Diccionario Maya Itzaj-Español-Ingles. Salt Lake City: University of Utah Press.

Hofling, Charles Andrew, with Félix Fernando Tesucún. 2000. Itzaj Maya grammar. Salt Lake City: University of Utah Press.

Holt, Dennis. 1999. Tol (Jicaque). Munich: Lincom Europa.

Hovdhaugen, Even. 2004. Mochica. Munich: Lincom Europa.

Holmer, Nils M. 1963. Gramatica comparada de un dialecto del Choco (con textos, índice y vocabulario. Etnologiska Studier 26: 79-248.

Ixchajchal Batz, Estanislao Augusto, Luis Mateo Cumez, and Candelaria Dominga López Ixcoy. 1996.Gramatica del idioma k’iche’. La Antigua Guatemala: Proyecto Lingüístico Francisco Marroquín.

Jacobsen J., William Horton. 1964. A grammar of the Washo language. Dissertation, University of California at Berkeley.

Jacobsen, Jr., William H. 1996. Beginning Washo. Carson City: Nevada State Museum.

Jany, Carmen. 2009. Chimariko grammar: areal and typological perspective. Berkeley /Los Angeles: University of California Press.

Johnson, Heidi Anna. 2000. A grammar of San Miguel Chimalapa Zoque. Dissertation, University of Texas at Austin.

Jung, Ingrid. 2008. Gramática del páez o nasa yuwe. Descripción de una lengua indígena de Colombia.Munich: Lincom Europa.

Kendall, Daythal L. 1977. A syntactic analysis of Takelma texts. Dissertation, University of Pennsylvania.

Kim, Yuni. 2008. Topics in the phonology and morphology of San Francisco del Mar Huave. Dissertation, University of California, Berkeley.

Kroeber, A. L. 1907. The Washo language of East Central California and Nevada. University of California Publications in American Archaeology and Ethnology 4 (5): 251-317.

Lai, I-Wen. 2009. Time in the Iquito language. Dissertation, University of Texas at Austin.

Larsen, Thomas Walter. 1988. Manifestations of ergativity in Quiché grammar. Dissertation, University of California, Berkeley.

LeCron Foster, Mary. 1969. The Tarascan Language. Berkeley/Los Angeles: University of California Press.

Leer, Jeffrey A. 1991. The schetic categories of the Tlingit verb. Dissertation, University of Chicago.

Lehmann, Walter. 1920. Zentral-Amerika. Teil 1: Die Sprachen Zentral-Amerikas in ihren Beziehungen zueinander sowie zu Süd-Amerika und Mexiko. Berlin: Verlag Dietrich Reimer (Ernst Vohsen).

Lindskoog, John N., and Ruth M. Brend. 1962. Cayapa phonemics. In: Elson (ed.), 31-44.

Lindskoog, John N., and Carrie A. Lindskoog. 1964. Vocabulario Cayapa. Quito: Instituto Lingüístico de Verano en cooperación con el Ministerio de Educación Publica.

Lugo, Bernardo de. [1619]1978. Gramática de la lengua general del Nuevo Reyno, llamada mosca. Madrid: Ediciones Cultura Hispánica del Centro Iberoamericano de Cooperación.

Maddieson, Ian, Heriberto Avelino, and Loretta O'Connor. 2009. The phonetic structures of Oaxaca Chontal. International Journal of American Linguistics 75 (1): 69-101.

Marlett, Stephen A. 1981. The structure of Seri. Dissertation, University of California:

Matras, Yaron, and Jeanette Sakel. 2007. Investigating the mechanisms of pattern

replication in language convergence. Studies in Language 31 (4): 829–865.

McQuown, Norman A. 1941. La fonémica del Cuitlateco. El México Antiguo 5: 239-254.

Mejía Fonnegra, Gustavo. 2000. Presentación y descripción fonológica y morfosintáctica del

waunana. In: González de Pérez and Rodríguez de Montes (eds.), 85-96.

Mixco, Mauricio Jesús. 1971. Kiliwa grammar. Dissertation, University of California at Berkeley. Mixco, Mauricio J. 1985. Kiliwa dictionary. Salt Lake City: University of Utah.

Mixco, Mauricio J. 2000. Kiliwa. Munich: Lincom Europa.

Moser, Mary , and Esteban Marlett. 1994. El desarrollo de clases nominales en seri. In: Gerardo López Cruz and José Luis Moctezuma Zamarrón (eds.): Estudios de lingüística y sociolingüística, 97-105. Hermosillo: Departamento de Letras y Lingüística, División de Humanidades y Bellas Artes, Universidad de Sonora, Instituto Nacional de Antropología e Historia.

Moser, Mary Beck, with the collaboration of Stephen Marlett. 1995. Seri de Sonora. Mexico, D.F.: El Colegio de México.

Moser, Mary Beck, and Stephen A. Marlett. 2004. Comcáac quih yaza quih hant ihíip hac. Cmiique Iitom-Cocsar Iitom-Maricáana Iitom. Diccionario seri-español-inglés. Con índices español-seri, inglés-seri, y con gramática. Hermosillo: Universidad de Sonora, Maestría en Lingüística, Centro de las Artes, Rosales y Luis Donaldo Colosio/ México, D.F.: Plaza

Naish, Constance M. 1979. A syntactic study of Tlingit. Dallas: Summer Institute of Linguistics.

Nevin, Bruce Edwin. 1998. Aspects of Pit River phonology. Dissertation, University of Pennsylvania.

Newman, Stanley. 1944. Yokuts language of California. New York: The Viking Fund.

O’Connor, Loretta. 2007. Motion, transfer and transformation. The grammar of change in Lowland Chontal. Amsterdam/Philadelphia: John Benjamins.

O’Connor, Loretta, and Peter C. Kroefges. 2008. The land remembers: landscape terms and place names in Lowland Chontal of Oaxaca, Mexico. Language Sciences 30 (2/3): 291-315.

Ocampo, Francisco Antonio. 1982. The phonology of Shelknam: the segmentals. MA thesis, State University of New York at Buffalo.

Oliverio, Giulia R.M. 1996. A grammar and dictionary of Tutelo. Dissertation, University of Kansas.

Ostler, Nicholas. 1993. Cases, directionals and conjunctions in Chibcha. Estudios de Lingüística Chibcha 12: 7-33.

Ostler, Nicholas. 1994. Syntactic typology of Muisca – a sketch. In: Peter Cole, Gabriella Hermon and Mario Daniel Martín (eds.), Language in the Andes, 205-30. Newark, Delaware: Latin American Studies Program, University of Delaware.

Overall, Simon E. 2007. A grammar of Aguaruna. Dissertation, La Trobe University.

Quirós Rodríguez, Juan Santiago. 1985. Observaciones sobre la tipología de la lengua chorotega o mangue. Filología y Lingüística 11 (2): 93-97.

Parker, Gary John. 1969. Ayacucho Quechua grammar and dictionary. The Hague: Mouton.

Payne, Doris Lander. 1985. Aspects of the grammar of Yagua: a typological approach. Dissertation, University of California, Los Angeles.

Payne, Doris L., and Thomas E. Payne. 1990. Yagua. In: Desmond C. Derbyshire and Geoffrey K. Pullum (eds.): Handbook of Amazonian Languages, vol. 2, 249-474. Berlin/New York/Amsterdam: Mouton de Gruyter.

Peña, Jaime German. 2015. A grammar of Wampis. Dissertation, University of Oregon.

Pérez Alonso, Juventino de Jesús et al. N.d. Pujb'il Yol Mam. Diccionario bilingüe Mam-Español. Noplace: Academia de lenguas Mayas de Guatemala.

Peyró García, Miguel. 2005. Estructuras gramaticales en el glosario de la lengua atacameña (1896). Liames 5: 25-42.

Pickett, Velma. 2007. Vocabulario Zapoteco del Istmo. Español-zapoteco y zapoteco-español. 5th ed.Tlalpan, D.F.: Instituto Lingüístico de Verano.

Pickett, Velma B., Cheryl Black, and Vicente Marcial Cerqueda. 1998. Gramatica popular del Zapoteco del Istmo. Juchitán/Tuscon: Centro de Investigación y Desarrollo Binnizá/Instituto Lingüístico de Verano.

Pickett, Velma B., María Villalobos Villalobos, and Stephen A. Marlett. 2010. Isthmus (Juchitán) Zapotec. Journal of the International Phonetic Association 40 (3): 365-372.

Pitkin, Harvey. 1984. Wintu grammar. Berkeley/Los Angeles/London: University of California Press.

Pitkin, Harvey. 1985. Wintu dictionary. Berkeley/Los Angeles/London: University of California Press.

Popky, Donna. 1999. Oro Win: A descriptive and comparative outlook of an endangered language. MA thesis, University of Pittsburgh.

Quesada Pacheco, Miguel Ángel. 2008. Gramática de la lengua guaymí (ngäbe). Munich: LincomEuropa.

Radin, Paul. 1929. A grammar of the Wappo language. Berkeley University of California Press.

Rasch, Jeffrey Walter. 2002. The basic morpho-syntax of Yaitepec Chatino (Mexico). Dissertation, Rice University.

Ribeiro, Eduardo Rivail. 2012. A grammar of Karajá. Dissertation, University of Chicago.

Robins, R. H. 1958. The Yurok language: grammar, texts and lexicon. Berkeley/Los Angeles: University of California Press.

Rojas Berscia, Luis Miguel. 2014. A heritage reference grammar of Selk'nam. MA thesis, Radboud Universiteit Nijmegen.

Romero-Figeroa, Andrés. 1997. A reference grammar of Warao. Munich/Newcastle: LINCOM Europa.

Rosés Labrada, Jorge E. 2015. The Mako language: Vitality, grammar and classification. Dissertation, University of Western Ontario.

Sachse, Frauke. 2010. Reconstructive description of eighteenth-century Xinka grammar. Utrecht: LOT.

Sadowsky, Scott, Héctor Painequeo, Gastón Salamanca, and Heriberto Avelino. 2013. Illustrations of the IPA: Mapudungun. Journal of the International Phonetic Association 43 (1): 87-96.

Sáez Godoy, Leopoldo, et al. 1974. Diccionario Español-Kunsa, Kunsa-Español. Valparaíso: Universidad Católica de Valparaíso.

Salas García, José Antonio. 2002. Diccionario Mochica-Castellano. Lima: Universidad de San Martín de Porres, Escuela Profesional de Turismo y Hotelería.

Sapir, Edward. 1922. The fundamental elements of Northern Yana. University of California Publications in American Archaeology and Ethnology 13: 215-234.

Sapir, Edward. 1923. Text analyses of three Yana dialects. University of California publications in American archaeology and ethnology 20: 263-294.

Sapir, Edward, and Morris Swadesh. 1960. Yana dictionary. Ed. by Mary Haas. Berkeley/Los Angeles: University of California Press.

Sapir, Edward. 1990. Takelma texts and grammar Ed. by Victor Golla. Berlin/New York: Mouton de Gruyter.

Sapir, Edward. 2001. Northwest California linguistics. Ed. By Victor Golla and Sean O’Neill. Berlin/New York: Mouton de Gruyter.

Seler, Eduard. 1902. Die Sprache der Indianer von Esmeraldas. In: Gesammelte Abhandlungen zur Amerikanischen Sprach- und Alterthumskunde. Vol. 1: Sprachliches.Bilderschriften. Kalender und Hieroglyphenentzifferung, 49-64. Berlin: A. Asher & Co.

Shaul, David L. 1995. The Huelen (Esselen) language. International Journal of American Linguistics 61: 191-239.

Silver, Shirley. 1966. The Shasta language. Dissertation, University of California at Berkeley.

Sischo, William R. 1979. Michoacán Nahuatl. In: Langacker (ed.), 307-380.

Slocum, Marianna C., and Florence L. Gerdel. 1983. Diccionario páez-español, español-páez.Lomalinda/Meta: Editorial Townsend.

Smeets, Ineke. 2008. A grammar of Mapuche. Berlin/New York: Mouton de Gruyter.

Strom, Clay. 1992. Retuarã syntax. Dallas: The Summer Institute of Linguistics/University of Texas at Arlington.

Suárez, Jorge A. 1983. La lengua Tlapaneca de Malinaltepec. México: Universidad Nacional Autónoma de México.

Suttles, Wayne. 2004. Musqueam reference grammar. Vancouver: University of British Columbia Press.

Thiesen, Wesley, and David Weber. 2012. A grammar of Bora, with special attention to tone. Dallas: SIL International.

Thompson, Sandra A., Joseph Sung-Yul Park, and Charles N. Li. 2006. A reference grammar of Wappo. Berkeley/Los Angeles: University of California Press.

Tuggy, David H. 1979. Tetelcingo Nahuatl. In: Langacker (ed.), 1-140.

Turner, Paul Raymond. 1966. Highland Chontal grammar. Dissertation, University of Chicago.

Twitchell, X̱'unei Lance. 2016. Haa wsineix̱ haa yoo x̱'atángi. Our language saved us: a guidebook for learning the Tlingit language. Juneau/Fairbanks: Goldbelt Heritage Foundation/Alaska Native Language Center.

Valiñas Coalla, Leopoldo, Mario Cortina Borja, and Miguel Mireles Padilla. 1984. Notas sobre el Cuitlateco. Anales de Antropología 21 (1): 171-197.

Vallejos, Rosa. 2016. A grammar of Kukama-Kukamiria: A language from the Amazon. Leiden/Boston: Brill.

Van der Voort, Hein. 2004. A grammar of Kwaza. Berlin/New York: Mouton de Gruyter.

Vittadello, P. Alberto. 1988. Cha'palaachi. El idioma cayapa. Guayaquil: Pontificia Universidad Católica del Ecuador/Museos del Banco Central del Ecuador.

Weber, David John. 1989. A grammar of Huallaga (Huánuco) Quechua. Berkeley/Los Angeles/London: University of California Press.

Waterhouse, Viola Grace. 1962. The grammatical structure of Oaxaca Chontal. Bloomington: Indiana University Research Center in Anthropology, Folklore, and Linguistics.

Weber, David John, Félix Cayco Zambrano, Teodoro Cayco Villar, and Marlene Ballena Dávila. 1998. Rimaycuna. Quechua de Huánuco. Diccionario del quechua del Huallaga con índices castellano e inglés. Lima: Instituto Lingüístico de Verano.

Weigel, William Frederick. 2005. Yowlumne in the twentieth century. Dissertation, University of California at Berkeley.

Wheeler, Alva L. 1970. Grammar of the Siona language, Colombia, South America. Dissertation, University of California at Berkeley.

Wichmann, Søren. 2007. The reference-tracking system of Tlapanec: between obviation and switch-reference. Studies in Language 31 (4): 801-827.

Wichmann, Søren. 2009. Case relations in Tlapanec, a head-marking language. In: Andrej Malchukov and Andrew Spencer (eds.): The Oxford handbook of case, 797-807. New York: Oxford University Press.

Wiebe, Neil, and Ruth Wiebe. 2015. Cayapa dictionary. In: Mary Ritchie Key and Bernard Comrie (eds.): The intercontinental dictionary series. Leipzig: Max Planck Institute for Evolutionary Anthropology. http://ids.clld.org/contributions/245

Wilbert, Johannes. 1962. Notes on a Sanema vocabulary. Journal de la Société des Américanistes 51: 83-101.

Wipio Deicat, Gerardo. 1996. Diccionario Aguaruna-Castellano, Castellano-Aguaruna. Revised by Alejandro Paati Antunce Segundo and Martha Jakway. Lima: Instituto Lingüístico de Verano.

Wojtylak, Katarzyna I. 2017. A grammar of Murui (Bue): A Witotoan language of Northwest Amazonia. Leiden/Boston: Brill.

Woodbury, Hanni. 2018. A reference grammar of the Onondaga language. Toronto: University of Toronto Press.

Zamponi, Raoul. 2004. Fragments of Waikuri (Baja California). Anthropological Linguistics 46 (2): 156-193.

References:

Adelaar, W. Towards a typological profile of the Andean Languages. In Evidence and counter-evidence: essays in honour of Frederik Kortlandt, vol. 2: general linguistics (eds. Lubotsky, A., Schaeken, J., Wiedenhof, J., Derksen, R., & Siebinga, S.), 23–33 (Rodopi, 2008).

Adelaar, W.F.H., & Muysken, P.C. The languages of the Andes (Cambridge University Press, 2004).

Bickel, B. Large and ancient linguistic areas. In Language dispersal, diversification, and contact: a global perspective (eds. Crevels, M. & Musyken, P.) 78–101 (Oxford University Press, 2020).

Brown, C.H., Wichmann, S., & Beck, D. Chitimacha: a Mesoamerican language in the Lower Mississippi Valley. I.nt. J. Amer. Linguistics 80, 425–474.

Campbell, L. Why is it so hard to define a linguistic area? In The Cambridge Handbook of Areal Linguistics (ed. Hickey, R.) 19–39 (Cambridge University Press, 2017).

Dediu, D., & Cysouw, M. Some structural aspects of language are more stable than others: a comparison of seven methods. PLOS ONE 8, e55009 (2013).

Derbyshire, D.C. Morphosyntactic areal characteristics of Amazonian languages, Int. J. Amer. Ling. 53 (3): 311–326 (1987).

Dixon, R.M.W., & Aikhenvald, A.Y. Introduction. In The Amazonian languages (eds. Dixon, R.M.W, & Aikhenvald, A.Y.), 1–22 (Cambridge University Press, 1999).

Dryer, M.S. 1988. Object-verb order and adjective-noun order: dispelling a myth. Lingua 74,: 185–217.

Emlen, N.Q. The Quechuan-Aymaran relationship. In The Oxford Guide to the Languages of the Central Andes (ed. Urban, M.) (Oxford University Press, to appear).

Emlen, N.Q., Van Gijn, R., & Norder, S. The Andean-Amazonian interface: sociolinguistic relations and areal-typological patterns. In The Oxford Guide to the Languages of the Central Andes (ed. Urban, M.). (Oxford University Press, to appear).

Epps, P. Amazonian linguistic diversity and its sociocultural correlates. In Language dispersal, diversification, and contact: a global perspective (eds. Crevels, M., & Muysken, P.) 275–290 (Oxford University Press, 2020).

Fernández Garay, A. Difusión de rasgos en Patagonia. In IV Congreso International de Letras, 307–313 <http://eventosacademicos.filo.uba.ar/index.php/CIL/IV-2010/paper/view/2667>

Golla, V. California Indian languages. (University of California Press, 2011).

Green, R. Location of the Polynesian homeland: A continuing problem. in Studies in Pacific languages and cultures in honor of Bruce Biggs (eds. Hollyman, J. & Pawley, A.) 133–158 (Linguistic Society of New Zealand, Auckland, 1981).

Haas, M.R. The Northern California linguistic area. In Hokan studies: papers from the First Conference on Hokan Languages held in San Diego, California, April 23-25, 1970 (eds. Langdon, M. & Silver, S.) 347–359 (The Hague, 1976).

Hammarström, H., Forkel, R., Haspelmath, M., & Bank, S. Glottolog 4.8. (2023)

Haynie, H.J. Studies in the history and geography of California languages (University of California at Berkeley, 2014).

Heckenberger M. The Arawak diaspora. In The Oxford Handbook of Caribbean archaeology (eds.Keegan, W.F., Hofman, C.L. &Rodríguez Ramos, R.), 112–125 (Oxford University Press, 2013).

Heggarty, P. Linguistics for archaeologists: a case-study in the Andes. Cambridge Archaeol. J., 18, 35–56.

Irwin, G. The Prehistoric Exploration and Colonialisation of the Pacific. (Cambridge University Press, Cambridge, 1994).

Kirch, P. & Green, R. Hawaiki, Ancestral Polynesia: An Essay in Historical Anthropology. (Cambridge University Press, Cambridge, 2001). doi:10.1017/CBO9780511613678.

Le Roux, B., & Rouanet, H. Multiple correspondence analysis (Sage, 2010).

Michael, L., & Robertson, A. Central Andean segmental phonologies in continental sperspective. In The Oxford Guide to the Languages of the Central Andes (ed. Urban, M.) (Oxford University Press, to appear).

Muysken, P. (ed.) From linguistic areas to areal linguistics (John Benjamins, 2008).

Nerbonne, J., Heeringa, P, & Kleiweg, P.[Comparison and Classification of Dialects](https://aclanthology.org/E99-1048). In Ninth Conference of the European Chapter of the Association for Computational Linguistics, pages 281–282, Bergen, Norway. Association for Computational Linguistics. 1999.

Nichols, J. Linguistic diversity in space and time (University of Chicago Press, 1992).

Nichols, J., & Peterson D.A. The Amerind personal pronouns. Language, 72, 336–371 (1996).

Nichols, J., Witzlack-Macarevich, A., & Bickel, B. The Autotyp genealogy and geography database. 2013 Release. <https://www.autotyp.uzh.ch/download/release_2013/autotyp-release_2013.pdf>

Pawley, A. & Green, R. Dating the Dispersal of the Oceanic Languages. Oceanic Linguistics 12, 1–67 (1973).

Payne, D.L. Introduction. In Amazonian linguistics: studies in lowland South American languages (ed. Payne, D.L.), 1–10 (University of Texas Press, 1990).

Swadesh, M. Mosan I: a problem of remote common origin. Int. J. Amer. Linguistics, 19, 26–44 (1953).

Thomason, S.R. Linguistic areas and language history. Studies in Slavic and General Ling., 28, 311–327 (2000).

Thomason, S.R. The Pacific Northwest Linguistic Area: historical perspectives. In The Routledge Handbook of Historical Linguistics (eds. Bowern, C. & Evans, B.) 726–736 (Routledge, 2015).

Urban, M. Is there a central Andean linguistic area? A view from the perspective of the “minor” languages. J. Lang. Contact 12 (2): 271–30 (2019).

Urban, M., Reyes-Centeno, H., Bellamy, K., & Pache, M. The areal typology of western Middle and South America: towards a comprehensive view. Linguistics 52, 1403-1463 (2019).

Van Gijn, R. The Andean foothills and adjacent Amazonian fringe. In The native languages of South America: origins, development, typology (eds. O’Connor, L., & Muysken P.), 102–25 (Cambridge University Press, 2014).

Van Gijn, R. & Muysken, P. Highland–lowland relations: a linguistic view. In Rethinking the Andes–Amazonia divide: a cross-disciplinary exploration (eds. Pearce, A.J., Beresford-Jones,D.G., & Heggarty, P.), 178–210 (UCL Press, 2020).

Viegas Barros, J.P. Lingüística areal en la Patagonia. In: iii Encuentro de Lenguas Indígenas Americanas (ELIA). Libro de actas (eds. Malvestitti, M., & Dreidemie, P.), 585–598 (Universidad Nacional de Río Negro, 2014).
